# Targeting Bacterial Cell Wall Synthesis: Structural Insights and Emerging Therapeutic Strategies

**DOI:** 10.3390/pharmaceutics18010106

**Published:** 2026-01-13

**Authors:** Bharat Kumar Reddy Sanapalli, Christopher R. Jones, Vidyasrilekha Sanapalli

**Affiliations:** 1 Department of Pharmacology, School of Pharmacy and Technology Management, SVKM’s Narsee Monjee Institute of Management Studies (NMIMS) Deemed-to-be-University, Jadcherla, Hyderabad 509301, India; bharathsanapalli@yahoo.in; 2 Department of Chemistry, Queen Mary University of London, Mile End Road, London E1 4NS, UK; c.jones@qmul.ac.uk; 3 Department of Pharmaceutical Chemistry, School of Pharmacy and Technology Management, SVKM’s Narsee Monjee Institute of Management Studies (NMIMS) Deemed-to-be-University, Jadcherla, Hyderabad 509301, India

**Keywords:** antibiotic resistance, cell wall synthesis, peptidoglycan biosynthesis, crystal structures, drug discovery

## Abstract

The emergence of multidrug-resistant (MDR) bacterial pathogens has heightened the urgency for novel antibacterial agents. The bacterial cell wall usually comprises peptidoglycan, which presents a prime target for antibacterial drug development due to its indispensable role in maintaining cellular integrity. Conventional antibiotics such as β-lactams and glycopeptides hinder peptidoglycan synthesis through competitive binding of penicillin-binding proteins (PBPs) and sequestration of lipid-linked precursor molecules. Nevertheless, prevalent resistance mechanisms including target modification, β-lactamase hydrolysis, and multi-drug efflux pumps have limited their clinical utility. This comprehensive analysis explicates the molecular machinery underlying bacterial cell wall assembly, evaluates both explored and unexplored enzymatic nodes within this pathway, and highlights the transformative impact of high-resolution structural elucidation in accelerating structure-guided drug discovery. Novel targets such as GlmS, GlmM, GlmU, Mur ligases, D,L-transpeptidases are assessed for their inclusiveness for the discovery of next-generation antibiotics. Additionally, cell wall inhibitors are also examined for their mechanisms of action and evolutionary constraints on MDR development. High-resolution crystallographic data provide valuable insights into molecular blueprints for structure-guided optimization of pharmacophores, enhancing binding affinity and circumventing resistance determinants. This review proposes a roadmap for future innovation, advocating for the convergence of computational biology platforms, machine learning-driven compound screening, and nanoscale delivery systems to improve therapeutic efficacy and pharmacokinetics. The synergy of structural insights and cutting-edge technologies offers a multidisciplinary framework for revitalizing the antibacterial arsenal and combating MDR infections efficiently.

## 1. Introduction

**Figure 1:**
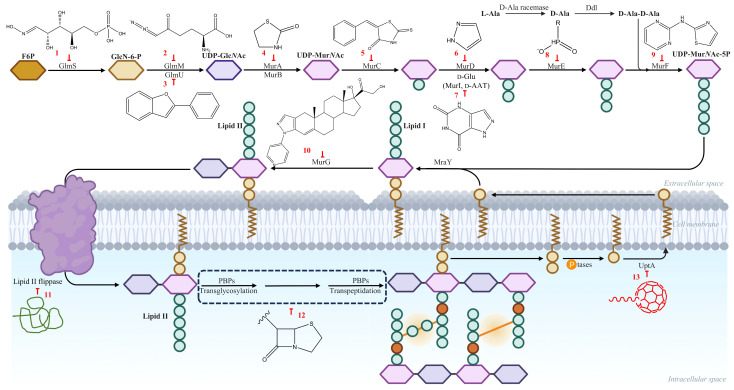
Schematic overview of bacterial peptidoglycan biosynthesis highlighting representative pharmacophore scaffolds mapped to specific enzymatic targets across cytoplasmic, membrane-associated, and periplasmic stages. The numbered chemical scaffolds indicate (1) arabinose oxime-5-phosphate targeting GlmS (2) 6-diazo-5-oxo-L-norleucine targeting GlmMand; (3) phenylbenzofurans targeting GlmU; (4) 4-thiazolidinones targeting MurB; (5) benzylidene rhodanines targeting MurC; (6) pyrazolyl derivatives targeting MurD; (7) pyrazolyl-pyrimidinediones targeting MurI/D-amino acid aminotransferases; (8) phosphinate analogues targeting MurE; (9) thiazolylaminopyrimidines targeting MurF; (10) murgocil targeting MurG; (11) phage lysis protein inhibiting lipid II flippase (MurJ); (12) β-lactams targeting penicillin-binding proteins (PBPs) involved in transglycosylation and transpeptidation; and (13) lipopeptides interfering with bactoprenol recycling and membrane-associated processes. Together, the figure illustrates how distinct chemical scaffolds engage multiple nodes of cell wall assembly.

The increasing prevalence of antibiotic resistance (ABR) has emerged as one of the most pressing public health threats of the 21st century [1,2]. The significant dissemination of multidrug-resistant (MDR) strains and the alarming rate at which resistance genes are emerging demands prompt action for the discovery of novel antibacterial targets and treatment approaches [3]. Targeting the cell wall is one of the most deep-rooted and successful approaches in antibacterial drug development. The unique components of the cell wall, including peptidoglycan that is lacking in human counterparts, provides a selective target that is critical for bacterial growth, survival and pathogenesis. It also obliges multiple essential roles including preserving cellular integrity and shape, osmotic lysis protection, and serving as a point of attachment for virulence factors and other surface proteins. The peptidoglycan is a macromolecular mesh usually composed of glycan strands cross-linked by short peptides. It envelops the cytoplasmic membrane and confers rigidity while allowing dynamic remodelling to house cell growth and division. The peptidoglycan layer in Gram positive bacteria (GPB) is thicker than in the Gram negative bacteria (GNB), but the latter are shielded by additional layer of OM [4]. The peptidoglycan backbone of the bacterial cell wall is made up of repeating units of *N*-acetylglucosamine (NAG) connected with *N*-acetylmuramic acid (NAM) via (1 → 4) glycosidic linkages; these together are flanked with pentapeptides (l-Ala–d-Glu–l-Lys–d-Ala–d-Ala) of NAM. The main difference in the cell walls of GNB from GPB is the diaminopimelic acid instead of l-lysine at the third position of a pentapeptide stem; however, additional structural variations and modifications of the peptide stems are known among different bacterial species. Beyond the classical GPB and GNB classification, acid-fast bacteria such as *Mycobacterium* species possess highly complex and expansively altered peptidoglycan–arabinogalactan–mycolic acid cell envelope architectures. The cell wall biogenesis itself acts as a target for many of the antibacterial agents. The synthesis of the cell wall involves both explored and unexplored enzymes that are essential for bacterial survival. To facilitate an intuitive understanding of how representative chemical scaffolds map onto distinct enzymatic targets in bacterial cell wall biosynthesis, a schematic overview is provided in Figure 1, while a detailed target-wise correlation of pharmacophores, structural data, and representative PDB entries is summarised in Table 1. Furthermore, information about different drugs that target cell wall synthesis, including their discovery or approval are depicted in Table 2. Biosynthesis of the cell wall has been categorized into three stages, namely cytoplasmic, membrane-associated and extra cytoplasmic stages [5]. These steps are catalysed by specialized enzymes, many of which are essential for bacterial viability, making them attractive candidates for antimicrobial intervention [4].

**Figure 2:**
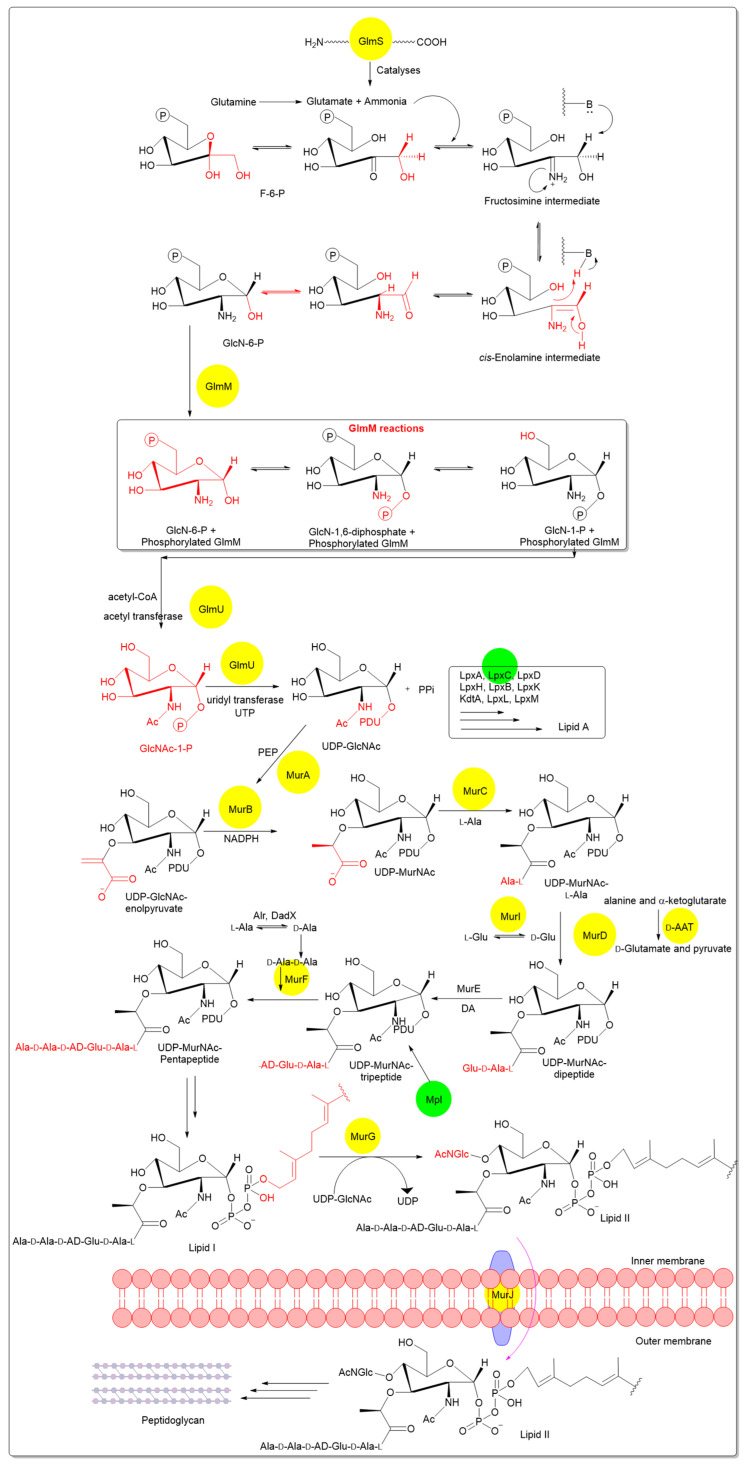
GlmS catalysed ketose/aldose isomerization. Fructose-6-phosphate reacts with ammonia to form fructosimine intermediate which eventually leads to cis-enolamine by stereospecific abstraction of the pro-R-proton of C-1. Cis-enolamine then undergoes reprotonation at the re face of the C-2 sp2 carbon to form glucosamine-6-phosphate. GlmM catalyses glucosamine-6-phosphate (Glc*N*-6-P) to glucosamine-1-phosphate (Glc*N*-1-P) by Ping-Pong Bi-Bi-mechanism. GlmU catalyzes the formation of UDP-N-acetylglucosamine (UDP-Glc*N*Ac) which eventually leads to peptidoglycan using Mur ligases (MurA-F), d-AAT, MurG, Mpl, MurJ. LpxC is zinc-dependent metalloamidase, catalyses lipid A biosynthesis, confined to Gram-negative bacteria. Yellow circles denote unexplored targets in all bacteria, and Green circles indicate unexplored targets confined to GNB.

Peptidoglycan biosynthesis begins in the cytoplasm, where UDP-*N*-acetylglucosamine (UDP-Glc*N*Ac) is synthesized from fructose-6-phosphate via enzymes GlmS, GlmM, and GlmU. UDP-Glc*N*Ac is converted to UDP-*N*-acetylmuramic acid (UDP-Mur*N*Ac) by MurA, the first committed step [6]. Subsequent ATP-dependent ligases (MurC–MurF) and auxiliary enzymes (Alr, Ddl) add amino acids to form UDP-Mur*N*Ac-pentapeptide. Despite their conservation across bacteria, no FDA-approved drugs target GlmS/M/U, though Mur ligases and d-Ala-d-Ala ligase (Ddl) are explored for broad-spectrum inhibitors [7]. In the membrane-associated stage, MraY transfers MurNAc-pentapeptide to undecaprenyl phosphate, forming Lipid I, which MurG modifies to Lipid II by adding GlcNAc. Lipid II is flipped to the periplasm by MurJ (or related flippases). The extra-cytoplasmic stage involves polymerization of Lipid II into glycan strands by penicillin-binding proteins (PBPs), with transglycosylases elongating chains and transpeptidases cross-linking peptides [8,9]. The cytoplasmic stages of peptidoglycan precursor biosynthesis, including key enzymatic steps, chemical intermediates, and representative points of pharmacological intervention, are schematically illustrated in Figure 2.

Given the centrality of peptidoglycan biosynthesis to bacterial physiology, it is not surprising that many of the most successful antibiotics, including β-lactams, glycopeptides, and phosphonic acid derivatives, act by targeting this pathway [10]. β-lactams such as penicillins, cephalosporins, and carbapenems function by acylating the active site serine of PBPs, thereby inhibiting transpeptidation. Furthermore, lipoglycopeptides like vancomycin, teicoplanin, and dalbavancin bind to the d-Ala-d-Ala moiety of the peptide, and inhibit the access of substrates to PBPs. These glycopeptides target enzymes or substrates that are unique to, or only found in, bacteria and not in humans, thereby reducing the risk of off-target toxicity [11,12]. Nonetheless, off-target inhibition has been reported for certain cell wall biosynthesis inhibitors; for example, MraY inhibitors have been shown to inhibit mammalian GPT enzymes involved in protein N-glycosylation, due to similarities in the catalytic reactions they mediate [13].

Despite the past efficacy of these agents, ABR has been rapidly evolving. Resistance mechanisms include the production of β-lactamases that degrade certain β-lactams, alterations in PBPs that reduce binding of the drugs (methicillin-resistant *S. aureus*), modification in precursors and overexpression of efflux pumps or porin mutations in GNB [14]. The dynamic emergence of resistance highlights the necessity for further investigation of additional targets in the cell wall biosynthetic pathway and for the development of novel inhibitors with different mechanisms of action.

In the recent past, there has been increased focus on the earlier, and less-characterized, steps of the peptidoglycan biosynthetic pathway, such as the Mur ligases and enzymes responsible for the bio- and recycling of the lipid carrier [15]. These enzymes are indispensable for bacteria viability and lack human homologs, which makes them very appealing from a drug discovery point of view; however, they are not currently addressed by any clinically approved antibiotic. So far, structural analyses have started to reveal the structure and catalytic mechanism of these proteins, thereby forming the basis for a rational drug design. Furthermore, the recent identification of new natural antibacterial hit compounds such as teixobactin, which targets lipid II and lipid III precursors, has rekindled an interest in this field, showing that it is feasible to develop antibiotics featuring novel scaffolds and a low potential for resistance [16]. Teixobactin additionally targets periplasmic-facing C55P and C55PP, thereby disrupting lipid carrier recycling and cell wall biosynthesis [17]. Besides the existing literature on the biosynthesis of peptidoglycan and cell wall-active antibiotics, a number of recent reviews on the topic have identified new strategies and structural understanding in this field. As one example, Garde and colleagues (2021) gave a new summary of the peptidoglycan structure, biosynthetic pathways and regulatory circuits by highlighting how cell wall plasticity supports intrinsic and acquired resistance [18]. In more recent studies, Torrens et al. (2024) described the mechanisms through which bacterial cell wall variability and adaptivity are conferred and associated peptidoglycan remodelling with survival in antibiotic stress and host-imposed challenges [19]. Supplementary to these biological approaches, Bagdad et al. (2024) summarised the application of cheminformatics and artificial intelligence to antibacterial discovery with case studies showing findings of novel small-molecule scaffolds with activity against drug-resistant pathogens by machine learning and computer-aided design [20]. The combination of these new works places the current study in the context of the changing research environment and highlights the life-long relevance of diversifying the antibacterial armamentarium through peptidoglycan-directed and AI-enabled discovery approaches.

The integration of advanced technologies such as high-throughput screening, structure-based drug design, and artificial intelligence in medicinal chemistry is poised to accelerate the discovery of next-generation antibiotics [21]. Structural biology, particularly X-ray crystallography and cryo-electron microscopy, plays a pivotal role in this endeavour by providing detailed snapshots of target proteins and their complexes with ligands. While numerous enzyme–inhibitor complex structures are available, relatively few peptidoglycan membrane enzymes have been captured in complex with their native polyprenyl-linked substrates, largely due to the conformational flexibility and hydrophobicity of these ligands. These insights facilitate the optimization of binding interactions and pharmacokinetic properties, thereby improving efficacy and reducing toxicity. In summary, bacterial cell wall synthesis remains a fertile ground for antibiotic discovery. While traditional targets have yielded potent therapeutics, the growing tide of resistance necessitates diversification of strategies. By expanding the repertoire of targets and leveraging structural insights, it is possible to develop novel, effective, and durable antibacterial agents capable of countering the threat of MDR pathogens [22]. This review aims to provide a comprehensive overview of both classical and emerging targets within the bacterial cell wall biosynthetic pathway, highlighting their structural features, inhibitor development, and potential in therapeutic innovation.

## 2. Established and Clinically Exploited Targets in Bacterial Cell Wall Biosynthesis

The intricate and highly conserved pathway of bacterial peptidoglycan biosynthesis has long served as a cornerstone of antibacterial drug discovery [23]. Several enzymes along this pathway have been identified as potential drug targets, primarily due to their pivotal roles in bacterial survival and a lack of homologous targets in humans. Among these, the PBPs and other proteins associated with the cytoplasm and membranes have been well examined, structurally defined, and targeted by multiple antibiotics.

### 2.1. Periplasmic Cross-Linking Enzymes

PBPs are enzymes that play a key role in peptidoglycan synthesis in bacteria. PBPs catalyze two processes: transglycosylation (polymerization of glycan strands) and transpeptidation (cross-linking of peptide chains) [24]. Both high molecular weight (HMW) and low molecular weight (LMW) forms of PBPs exist, and the HMW PBPs are directly responsible for these fundamental processes leading to bacterial cell wall formation [18]. Transpeptidase activity of PBPs is the primary target of β-lactams, which forms a covalent linkage with the serine residue at the active site of PBPs, resulting in irreversible inactivation, interfering with cell wall synthesis and causing bacterial lysis. The effectiveness of β-lactams is mainly due to a high degree of specificity for PBPs [25]. However, different mechanisms of resistance have reduced their efficacy, including β-lactamase production, modification of PBPs, and efflux pumps. To overcome these challenges, β-lactamase inhibitors such as clavulanic acid, tazobactam, and avibactam have been co-administered with β-lactams, restoring their activity against resistant strains. Structural studies have provided detailed insights into the binding interactions of these inhibitors with their respective enzymes, informing the rational design of next-generation derivatives.

Transglycosylase and transpeptidase domains of PBPs continue to be prime targets due to their indispensable roles in polymerizing and cross-linking glycan strands [26]. Novel β-lactams such as ceftolozane and ceftazidime/avibactam have been developed to target resistant PBPs with improved binding affinity. Glycopeptides are another class of antibiotics that act on the d-Ala-d-Ala peptide termini; however, resistance has followed and that has prompted the search for lipoglycopeptides (dalbavancin, telavancin, and oritavancin), thereby widening focus to new derivatives that have more potent interactions and superior membrane anchoring. Moenomycin is a natural compound that directly targets the transglycosylase activity of PBPs, thereby inhibiting glycan chain polymerization. Despite its activity of potency, limited oral bioavailability and pharmacokinetics have restricted its clinical development. Nevertheless, moenomycin is an important lead compound for the development of new transglycosylase inhibitors. Recent research has focused on the synthesis of moenomycin analogs that exhibit improved stability and bioavailability [27]. The derivatives exhibit promising in vitro activity and provide structural templates for the rational design of drugs.

### 2.2. Cytoplasmic Peptide Stem Synthesis Enzymes

The drug cycloserine is a structural analogue of d-Ala and a competitive inhibitor of alanine racemase and d-Ala-d-Ala ligase. These enzymes are crucial for the synthesis of the d-Ala-d-Ala dipeptide in the pentapeptide stem of precursors of peptidoglycan. Inhibition of these enzymes by cycloserine blocks the completion of the stem peptide, and thus the structural integrity of peptidoglycan is lost. Although effective, cycloserine is neurotoxic at therapeutic doses, thus limiting its use mostly to second-line treatment of drug-resistant tuberculosis. Its mechanism of action and unusual target make it a useful model for future drug development [28].

### 2.3. Lipid Carrier Recycling and Membrane-Associated Targets

Bacitracin inhibits the removal of the terminal phosphate group from undecaprenyl diphosphate (C55PP), thereby preventing regeneration of undecaprenyl phosphate (C55P). Sequestering the lipid carrier inhibits recycling that is required for ongoing cell wall synthesis. Used locally primarily because of nephrotoxicity, bacitracin is still effective in preventing Gram-positive dermal infections. Resistance mechanisms involving the Bcr transporter and target modification require careful use and surveillance [29]. In contrast to canonical target modification mechanisms, bacitracin resistance frequently arises from antibiotic-induced upregulation of redundant pyrophosphatases, including UppP, PgpB, LpxT, and YbjG, which rapidly convert C55PP to C55P, thereby reducing bacitracin binding [30].

### 2.4. Cell Wall Remodeling Enzymes

Amidases and carboxypeptidases that play a role in maturation and remodelling of cell walls are also possible targets but with less stringent investigation for therapeutic use [31]. They typically work with redundant peptide stems and control cross-linking to ensure cell wall strength and bacterial growth. Though their functions are dispensable under specific growth conditions, they are essential in particular stages of the life cycle and in precise environmental niches, representing provisional liabilities.

### 2.5. Structural Basis of Target Exploitation

Overall, the tapped targets in this pathway are still indispensable for antimicrobial intervention [32]. Their structural elucidation by cryo-EM, X-ray crystallography, and NMR spectroscopy has provided important insights into enzyme catalysis and drug binding. Such an abundance of structural information affords rational therapy design and allows for the creation of new inhibitors with improved specificity, potency, and resistance-evading properties. Ongoing refinement of known targets and creation of inhibitors specifically designed to target conserved structural motifs will play a significant role in the forward momentum of antibiotic therapy.

For clarity, the major stages of bacterial cell wall biosynthesis and their established molecular targets are summarized schematically in Figure 1.

## 3. Unexplored Targets in Cell Wall Biosynthesis

Though the greatest effort has been directed towards the peptidoglycan synthesis enzymes and intermediates, other regulatory processes and molecular players governing the cell wall synthesis have yet to be thoroughly investigated. These untapped targets are newer prospects for antibacterial drug development, mostly in the context of emerging AMR. Expanding the scope to more atypical targets like PBPs, researchers can look for newer vulnerabilities in the bacterial cell machinery. This section describes potential unexplored or under-investigated potential targets involved in biosynthesis, regulation, transport, and non-canonical maintenance of the cell wall, which can be potential sources for next-generation therapeutics [33].

### 3.1. Glucosamine-6-phosphate Synthase (l-glutamine: d-fructose-6-phosphate Amidotransferase; GlmS)

GlmS converts fructose-6-phopshate (F-6-P) into glucosamine-6-phosphate (Gl-6-P) using ammonia and glutamine by forming two intermediates, fructosamine-6-phopshate and *cis*-enolamine. Gl-6-P is eventually transformed into uridine diphospho-*N*-acetylglucosamine and many other amino sugar entities. Among these products, *N*-acetylglucosamine is a major constituent of the peptidoglycan layer of the bacterial cell wall [34,35,36]. Thus, GlmS offers a potential antibacterial target and has attracted the interest of research groups [35].

Two coupled enzymatic reactions are catalysed by GlmS. The first is glutamine hydrolysis, which produces glutamate and ammonia, both of which are transported to F-6-P. The second reaction is a Heyns rearrangement in which F-6-P is isomerized from a ketose to an aldose [37,38]. The crystal structure of GlmS reveals two distinct domains: an NH_2_-terminal, glutamine amidotransferase domain (catalyses glutamine hydrolysis) and a COOH-terminal synthase domain (catalyses glutamine isomerization) [39,40,41]. The studies by Corizzi et al., 1992, Badet-Denisot, 1995, Bearne, 1996 and Leriche, 1997 reported the importance of various sites and intermediates for the GlmS action. Site-directed mutagenesis (replacement of cysteine with alanine) reveals that the cysteine thiol at NH_2_-terminal is essential for the enzymatic activity of GlmS [42,43,44,45]. Hence, various studies were focussed on the inactivation of GlmS using glutamine affinity synthetic analogues such as 6-chloro-5-oxo-l-norleucine, 6-diazo-5-oxo-l-norleucine, γ-dimethylsulfonium and *N*^3^-fumaroyl l-2,3-diaminopropionic acid derivatives, which alkylate the essential cysteine residue [36,40,46]. Recently, researchers focused on the development of carbohydrate-based inactivators such as *N*-iodoacetylglucosamine-6-phosphate for increasing the specificity towards the glucosamine binding sites [38]. Derivatives of the *cis*-enolamine intermediate are expected to be inhibitors of the enzyme at the COOH terminal [43]. One such example, 2-amino-2-deoxy-d-glucitol 6-phosphate, is considered to be the most potent carbohydrate-based inhibitor reported to date [47]. Thus, two blocking sites, namely –NH_2_ terminal cysteine thiol and –COOH terminal sites, are to be thoroughly investigated for the effective inhibition of GlmS enzyme.

### 3.2. Phosphoglucosamine Mutase (GlmM)

GlmM is the key enzyme that plays a major role in the interconversion of Gl-6-P to glucosamine-1-Phosphate (Gl-1-P) by Ping-Pong Bi-Bi-mechanism; which eventually results in UDP-Glc*N*Ac. GlmM belongs to the superfamily of α-d-phosphohexomutases [8]. All these enzymes require Mg^2+^ ions for activity and a phosphoserine residue for phosphoryl transfer reaction [48]. GlmM usually contains four key regions in the catalytic pocket: phosphate-binding site (interactions between the product and phosphate group of substrates), a sugar-binding loop (residues bind with the sugar moiety of the product/substrate), the metal-binding site, and the phosphoserine residue (phosphoryl transfer reaction) [49].

Mengin-Lecreulx & van Heijenoort in 1996 reported the importance of GlmM in both lipopolysaccharide and peptidoglycan synthesis in *E. coli* by performing mutational studies [50,51]. In addition, GlmM and its homologs have been identified in *Helicobacter pylori* [52], *Staphylococcus aureus* [53], and *Pseudomonas aeruginosa* [54]. In 2008 Shimazu et al. also identified the GlmM homolog in *Streptococcus gordonii* using gene encoding methodology and studied its impact on cell morphology, growth, biofilm formation, and sensitivity to antibiotics in bacteria [55]. Currently, there is no existing crystal structure for the GlmM enzyme. Dai et al. (1992) discovered the crystal structure of rabbit muscle phosphoglucomutase, another enzyme of the hexosephosphate mutase family [56]. With this phosphoglucomutase, the predicted volume of the active site cleft containing the catalytic serine residue is large enough to accommodate an ATP molecule. So far, very few inhibitors have been discovered against GlmM, such as 2-azido-2-deoxy-α-d-glucopyranosyl phosphate and 2-amino-2,3-dideoxy-3-fluoro-α-*d*-glucopyranosyl phosphate. Similarly, the analogues of glucosamine-6-phosphate (Glc*N*-6P) were also anticipated to be inhibitors of GlmM [57].

### 3.3. N-Acetylglucosamine-1-phosphate Uridyl Transferase (GlmU)

GlmU is the third enzyme in the series of peptidoglycan synthesis, catalysing the acetyl group transfer from acetyl-CoA to Gl-1-P to generate *N*-acetylglucosamine-1-phosphate (Glc*N*Ac-1-P) [58,59]. Further, it also aids in the transfer of uridyl monophosphate from UTP to Glc*N*Ac-1-P to generate UDP-Glc*N*Ac and pyrophosphate [59]. The crystal structure of GlmU contains two distinct domains which are connected by an α-helix. The COOH and NH_2_ terminal domains of the enzyme are involved in the acetyltransferase and uridyltransferase activity, respectively [60]. In 2009, Brown et al. identified the first crystal structure of GlmU in *E. coli* [61]. Although crystal structures are available for most organisms, novel drug design and development has only progressed extensively in *Mycobacterium tuberculosis* (Mtb) and the enzyme is considered by the research community to be the most potent anti-TB target. Recent reports exemplified the inhibitory activities of GlmU using different compounds. Tran et al. reported in 2013 that aminoquinazoline-based molecules targeted the uridyltransferase activity of GlmU with an IC_50_ of 74 μM [62]. Soni et al. (2015) reported the uridyltransferase inhibitory activity of Oxa33 with an IC_50_ of 9.96 μM against Mtb H37Rv strain [63]. In addition, computational strategies were also attempted to discover the lead molecule against GlmU. In 2016 Mehra et al. screened 20,000 molecules against Mtb GlmU. Amongst them, the lead compound 5810599 was found to be effective in inhibiting the GlmU (IC_50_ of 9.018 μM) [64]. Furthermore, Rani et al. (2015) discovered four molecules that were active against acetyltransferase (IC_50_ 9–70 μM) of drug-resistant Mtb [65]. However, the investigation of GlmU in non-mycobacterial species has been notably limited. Consequently, there exists considerable potential for further exploration of this enzyme across various bacterial taxa beyond mycobacteria.

### 3.4. Mur Ligases

Mur ligases (MurA, MurB, MurC, MurD, MurE and MurF) act as catalysts in the synthesis of uridine diphosphate-*N*-acetyl glucosamine (UDP-Glc*N*Ac), the precursor for macromolecules of bacterial cell walls such as lipopolysaccharides in GNB and teichoic acid in GPB [66]. Despite the development of several compounds against Mur ligases, the lack of clinical approval does suggest that this target remains relatively unexplored in terms of successful translation into approved therapies. While significant research effort has been dedicated to understanding Mur ligases and developing inhibitors, the absence of approved compounds indicates that there is still much to learn and uncover about the complexities of this target and its potential as a therapeutic avenue.

#### 3.4.1. MurA (UDP-GlcNAc Enolpyruvyl Transferase)

MurA facilitates the transfer of an enolpyruvate group from phosphoenol pyruvate (PEP) to UDP-GlcNAc, resulting in the release of phosphate. The mode of action of this process follows an addition-elimination mechanism. Specifically, Asp305 and Cys115 (numbering of *E. coli* MurA) catalyse the anti-addition of PEP on UDP-GlcNAc, resulting in the formation of a tetrahedral intermediate. Subsequently, *syn*-elimination occurs, leading to the production of UDP-GlcNAc enolpyruvate [67]. The crystal structure of MurA was studied in detail by Skarzynski et al., in the year 1996 [68]. MurA consists of two globular domains that are joined by a double-stranded linker: catalytic domain (residues 22–229) and C-terminal domain (residues 1–21 and 230–419). The primary structural configuration of each domain has a high degree of similarity, characterised by three internal helices arranged in parallel, encircled by three helices and three four-stranded β-sheets that are exposed to the surrounding solvent. Each domain has an approximate tripartite symmetry that connects secondary structural elements within it.

Presently, an antibiotic employed to precisely target MurA is Fosfomycin (discovered in 1969, obtained from *Pseudomonas syringae* and *Streptomyces* spp.). It is a PEP analogue that forms a permanent bond with Cys115 at the active site of MurA in *E. coli*. This interaction leads to the inactivation of the enzyme and ultimately results in the death of the cell. Nevertheless, there are many mechanisms that confer resistance to Fosfomycin, including alterations in cellular permeability, mutations in the enzyme, or enzymatic processes that render the antibiotic inactive. Yet, because of its unique mechanism of action and synergy with other drugs, it is a valuable tool for combination therapy [69]. Several covalent and noncovalent inhibitors have been developed. For example, Avenaciolide compounds derived from *Neosartorya fischeri* act as tetrahedral intermediate inhibitors, whilst quinazolinone analogues function as competitive inhibitors [70].

#### 3.4.2. MurB (UDP-N-acetylenolpyruvylglucosamine Reductase)

MurB is crucial for bacterial cell viability. MurB facilitates the specific reduction of UDP-GlcNAc enolpyruvate to UDP-MurNAc using the coenzyme Nicotinamide Adenine Dinucleotide Phosphate (NADPH). The enzyme is equipped with an active site that includes the Flavin Adenine Dinucleotide cofactor (FAD). The initial stage of the process entails the development of the FAD-MurB complex, which serves as a redox intermediate. Following the formation of the second NADPH-MurB complex, the FAD molecule is reduced by NADPH, resulting in the development of the intermediate FADH_2_-MurB. This occurs by the transfer of the H4 (pro-*S*) atom from NADPH to the nitrogen atom of FAD. After the release of NADP^+^, the enzyme interacts with UDP-Glc*N*Ac enolpyruvate. In the second step, the FADH_2_-MurB enzyme reduces UDP-Glc*N*Ac enolpyruvate by transferring hydrogen from its C-3 position. The enolate intermediate formed after the release of FAD is stabilised by the substrate’s carboxylic acid with the enzyme. The presence of water is necessary for the substrate to undergo isomerization and form the end product UDP-Mur*N*Ac [71].

Availability of crystal structures of MurB from *E. coli* [71,72,73], *S. aureus* [74] and *S. pneumonia* [75] prompted the development of inhibitors using structure-based approaches [76]. In 1997, Benson et al. reported the crystal structure of MurB in complex with enolpyruvyl-UDP-N-acetylglucosamine (EP-UNAG), which led to the identification of key binding residues [72]. The two interactions between the carboxylate moiety and Arg159, Glu325, plus the diphosphate moiety and Tyr190, Ly217, Asn233 and Glu288 provides the transition state stabilization of the enzyme. Based on this insight into diphosphate binding, extensive research has been conducted to discover diphosphate mimetics as potent MurB inhibitors. Imidazolidinone was among the initial inhibitors to target the MurB site on *E. coli* that demonstrates efficacy against isolates of *S. aureus*. In 2000s Andres et al. reported that trisubstituted thiazolidinones acted as diphosphate surrogates for the substrate and served as potential *E. coli* MurB inhibitors with an IC_50_ of 7–28 μM [77]. In addition, Bronson et al. observed MurB inhibitory activity of imidazolidinone (IC_50_ of 12 μM) [78] in 2003.

#### 3.4.3. MurC-F (Synthetases)

MurC is an amide ligase whose activity is controlled in a phosphorylation-dependent manner in *S. pneumonia* and *Corynebacterium glutamicum.* MurC is involved in the production of the disaccharide-peptide moiety of peptidoglycan biosynthesis and catalyses the interconversion of UDP-MurNAc and UDP-MurNAc-Ala [79]. The crystal structures of MurC have been discovered in *E. coli* [80], *M. tuberculosis*, *M. leprae* [81], *P. aeruginosa* [82] and *Chlamydia tracomatis* [83]. The stereospecificity is absolute, as only the l-Ala (Gly and L-Ser in rare cases) is substrate but not d-Ala [80]. MurC possesses three domains: N-terminal domain (UDP binding domain), central domain (ATP binding domain) and C-terminal domain (ligand binding domain) [84,85]. The N-terminal domain binds to the UDP-*N*-acetylmuramic acid, the central and COOH domains interact with the ATP and incoming amino acids, respectively [86]. The COOH terminal domain is highly conserved amongst the enzymes of the Mur family as MurD share106 (RMSD of 2.2 Å), MurE 97 (2.6 Å) and MurF 107 (RMSD: 2.7 Å) equivalent Cα residues [85,87,88]. The COOH domain plays a crucial role in the catalytic mechanism of MurC; hence, its inhibition could provide valuable baseline for designing novel inhibitors [66,85]. The investigation into the inhibition of MurC using l-Ala analogues commenced prior to the complete elucidation of the enzyme’s structure. Takahashi and colleagues demonstrated that glycine acts as a competitive inhibitor, preventing the addition of l-Ala to the UDP-MurNAc substrate. Further studies conducted a few years later, including those by Liger et al. [80] and Emanuele et al., [89] provided evidence that certain l-amino acids, even without being incorporated into the natural substrate, may effectively hinder the activity of MurC. On the contrary, d configurations of amino acids failed to exhibit any inhibitory effect. In 2001 Reck et al. reported the inhibitory activity of phosphinate transition-state analogues against *E. coli* MurC with an IC_50_ of 49 nM [79]. Further, in 2008 Roman et al. reported *N*-acylhydrazones as potential inhibitors of MurC enzyme (IC_50_ of 123 μM) [90].

MurD facilitates the synthesis of UDP-Mur*N*Ac-l-Ala-*d*-Glu by catalysing the generation of an amide bond between d-Glu and UDP-Mur*N*Ac-l-Ala. Only prokaryotes metabolise amino acids with the D configuration, which is the primary distinguishing characteristic of the PG. This configuration likely confers resistance to destruction by external enzymes. Due to its exceptional specificity towards d-amino acid substrates, MurD has been recognised as a very attractive target for the development of selective antibacterial agents. The MurD reaction has been proposed as the point at which the synthesis of peptidoglycan (PG) is controlled to ensure the appropriate thickness of the PG layer in GNB [91]. The MurD crystal structures were extensively studied in *H. influenzae*, *E. coli*, *Enterococcus faecalis* and *S. aureus* [92,93,94]. Unlike the other Mur ligases, MurD is poorly specific towards UDP-precursor and 1-phospho-MurNAc-l-Ala acts as its substrate [94]. Bertrand et al. (1999) characterized the first crystal structure of *E. coli* MurD in complex with UDP-Mur*N*Ac-l-Ala [95]. The MurD crystal structure comprises three domains: The NH_2_ terminal domain (UDP-Mur*N*Ac-l-Ala binding), central domain (ATP binding) and COOH terminal domain (d-glutamic acid binding). The interactions of the substrate with NH_2_ terminal residues such as Leu15, Thr16, Asp35, Thr36, Arg37, Gly73, Asn138 and His183 provide greater stabilization of the enzyme; inhibition of which leads to the discovery of novel MurD inhibitors [96,97]. MurD inhibitors can be of two types: peptide-based and non-peptide-based inhibitors. Bratkovic et al. reported in 2008 that two cyclic nonapeptides comprising Cys-Ser-Ala-Trp-Ser-Asn-Lys-Phe-Cys and Cys-Pro-Ala-His-Trp-Pro-His-Pro-Cys, possess significant MurD inhibitory activity with IC_50_ of 0.62 and 1.5 mmol/L, respectively [98]. Many further reports exemplified the MurD inhibitory activity by non-peptide-based inhibitors such as 9H-Xanthene [99], *N*-Acylhydrazones [90], sulfonohydrazides [100], sulfonohydrazones [101], benzylidenethiazolidinones [102] and pulvinones [103].

MurE, the only Mur ligase with substrate specificity that differs between bacteria, adds the third residue to the PG peptide moiety. GNB, GPB, and Mycobacteria have a *meso*-diaminopimelic acid (mA_2_pm) residue at the third position of the PG peptide moiety, whereas GPB have an l-Lys residue [91]. Incorporation of the wrong amino acid may lead to bacterial cell lysis as evident from the report by Mengin-Lecreulx et al. in 1999 [104]. In addition to l-Lys and *meso*-A_2_pm, various analogues such as l-Orn, ll-A_2_pm, l-diaminobutyric acid, l-homoserine, cystathionine and lanothionine act as substrates for MurE [105,106,107]. Gordon et al. (2001) solved the crystal structure of *E. coli* MurE in complex with UDP-Mur*N*Ac-l-Ala-γ-d-Glu-*meso*-A_2_pm [87]. The crystal structure comprises three globular domains: the NH_2_ domain (1–88 residues) involved in binding of the UDP moiety, the central (90–338) and COOH domain (340–497). Among the three domains, two share similar topology reminiscent of the MurD three-dimensional structure. Further crystallographic analysis demonstrated that the presence of an invariant lysine residue is important in the inhibitory activity of MurD, -E and -F. The lysine residue in the form of carbamate derivative is crucial for acyl phosphate formation and Mg^2+^ binding. Various competitive inhibitors were reported against catalytic residues of MurE such as phosphinate derivatives [108] and diaminopimelic acid derivatives [106].

MurF enzyme catalyses the addition of dipeptide, d-Ala d-Ala at positions 4 and 5 of the peptide stem [109]. The dipeptide introduced by MurF plays a vital role in the construction of peptidoglycan (PG) by providing energy to the process for cross-linking glycan strands in the periplasmic region, where ATP is not present. MurF selectively employs dipeptide substrates consisting of d-amino acids and has a wider range of substrate acceptance compared to the other Mur ligases. MurF can incorporate non-standard d-amino acids, such as d-methionine, into the PG. Non-canonical d-amino acids are synthesised as a result of environmental stresses, such as the transition into stationary phase. The crystal structure of MurF enzyme comprises three domains: the NH_2_ domain (1–81 residues), central (92–310 residues) and COOH domain (311–447 residues). Aminoalkylphosphinate derivatives are the only reported phosphinate inhibitors of MurF to date. These aminoalkylphosphinate derivatives destabilize the transition state by competitively binding with the *E. coli* MurF enzyme [110].

### 3.5. Mpl

Murein peptide ligase (Mpl) is a non-essential enzyme that mostly presents in GNB. Mpl is involved in peptidoglycan recycling by adding tripeptide l-Ala-γ-d-Glu-meso-A_2_pm onto the Mur*N*Ac. The crystal structure has been resolved in *E. coli* and found to be homologous with MurC enzyme [111]. Though the enzyme is not essential, Mpl may serve as a potential antibacterial target [112]. Indeed, its broad specificity increases the probability of incorporating toxic peptides into growing peptidoglycan. To date, no small molecule inhibitor has been reported against Mpl except tri, tetra and pentapeptide substrates.

### 3.6. Glutamate Racemase (MurI) and d-Amino Acid Amino Transferase (d-AAT)

*d*-Glutamic acid is an important component of poly-γ-glutamate in some species of bacteria. The synthesis of glutamate is catalysed by two distinct enzymes: glutamate racemase (MurI) and d-amino acid amino transferase (*d*-AAT).

Glutamate racemase or MurI catalyses the interconversion of *l*- and *d*-enantiomers of glutamate. The MurI or RacE genes were extensively studied and it was concluded that all organisms except *Bacillus anthracis* (comprising the RacE1 and RacE2 gene) possess only one glutamate racemase gene. Further biochemical studies showed that *E. coli* MurI racemase requires UDP-Mur*N*Ac-l-Ala for its activation, which eventually leads to the formation of glutamic acid. MurI belongs to the amino acid racemase family, which operates without a cofactor or metal ion [113]. Several MurI crystal structures have been proposed in different organisms such as *E. coli* and *H. pylori*. In 2007, Lundqvist et al., reported a crystal structure of the MurI monomer consisting of two domains (NH_2_ and COOH) that exhibited a pseudo-symmetry axis which, along with conserved topology, was consistent with the reported *Bacillus subtilis* RacE crystal structure. The NH_2_ domain comprises conserved residues involved in d-glutamate deprotonation, while the COOH domain consists of catalytic residues involved in l-glutamate deprotonation. The two domains apparently move with respect to each other; however, the movement is largely restricted around a single axis, “hinge movement”. Since the concerted motion of the hinge occurs between two domain crossovers, binding site formation and catalysis need to be permitted. Moreover, the enzyme modulates its activity according to physiological requirements by controlling through independent mechanisms [114]. Therefore, MurI has also become a potential antibacterial target for the identification of new compounds. Due to its crucial role in the survival of bacteria, it is an ideal target for antibacterial agents. Ashiuchi and colleagues suggested in 1993 the use of l-serine O-sulphate as a suicidal substrate for MurI in *Pediococcus pentosaceus* [115]. Furthermore, Tanner et al. (1994) reported that aziridino glutamate irreversibly inactivates MurI in *Lactobacillus fermenti* via alkylation of a Cysteine residue in the catalytic pocket [116]. AstraZeneca discovered pyrazolopyrimidinedione as a non-competitive inhibitor of *H. pylori* MurI (IC_50_ = 1.4 μM). Although, the inhibitors exhibited promising in vitro activity, issues such as limited spectrum of activity, potential off-target effects, and sub-optimal pharmacokinetic properties necessitate the quest for novel inhibitors that can overcome these challenges and provide enhanced therapeutic benefits [114].

*d*-Amino acid aminotransferase (*d*-AAT) catalyses the conversion of alanine and α-ketoglutarate into d-glutamate and pyruvate. Because of its ubiquitous nature in most GPB, this enzyme can be used as a potential antibacterial target for the treatment of infections caused by resistant strains of GPB. Several crystal structures were resolved in different species like *Bacillus* and *Geobacillus*. In 1998 Sugio et al. reported that the crystal structure of *d*-AAT consists of two domains, an NH_2_ terminal or ‘small’ domain (1–120 residues, four-stranded antiparallel β-sheets and two long α-helices) and a COOH terminal of ‘large’ domain (121–282 residues, two mixed β-sheets) [117]. Both domains contribute to the formation of symmetrical active pockets, which will allow the attack of substrate.

### 3.7. MurG (Translocase II)

MurG, a membrane-associated glycosyltransferase, catalyses the transfer of Glc*N*Ac from UDP-Glc*N*Ac to the C4 hydroxyl of the membrane anchored Lipid I (undecaprenyl-pyrophosphate-Mur*N*Ac-pentapeptide, formed from UDP-Mur*N*Ac-pentapeptide by MraY enzyme, MurX in *Mycobacterium tuberculosis*) to form Lipid II [118]. Lipid II is a pivotal intermediate that links cytoplasmic and extracellular stages of peptidoglycan assembly. MurG’s essential role and absence in humans make it an attractive drug target. Structural studies have revealed a bilobal architecture with distinct donor and acceptor binding sites, enabling virtual screening for competitive inhibitors. In 2000 S. Ha et al. discovered the crystal structure of MurG at 1.9 Å in the *E. coli* by using multiple isomorphous replacements and an anomalous scattering technique [119]. The structure showed the existence of two distinct domains, separated by a deep cleft, and has an α/β open-sheet topology with a high degree of homology. The NH_2_ domain contains residues 7–163 and 341–357 with six α-helices and seven β-sheets. Alternatively, the COOH domain comprises residues 164–340 with eight α-helices and six parallel β-sheets, including one irregular bipartite helix (referred to as the α-link) connecting between the first β-strand of the COOH domain and the NH_2_ domain. Across both domains, the β-strands are coupled in a canonical Rossmann fold. In addition, the NH_2_ and COOH terminals are coupled by a short connector between the COOH domain α-link and NH_2_ domain β-strand. This interdomain linker and short peptide segment connecting the terminal helices of both the NH_2_ and COOH terminals form the bottom of the cleft separating the domains [119]. Drugs such as teixobactin, ramoplanin (Phase II clinical trials), enduracidin and mannopeptimycin were found to inhibit cell wall biogenesis by forming complexes with both lipids I and II or their intermediates [120]. However, nisin and mersacidin are the two drugs capable of recognizing the diphospho-sugar portion of Lipid II and exhibit antibacterial activity via pore-formation. Nisin was approved by the FDA to be used as a food preservative [121,122] and has exhibited resistance against most GPB by modifying the cell wall, drug inactivation and upregulating ABC transporters [123,124,125]. However, it should be noted that most glycopeptides are not direct inhibitors of MurG enzymes, as they do not permeate the cytoplasmic membrane, and their antibacterial activities are exhibited on the periplasmic side, where lipid-linked substrates are sequestered in order to inhibit further enzymatic reactions

The flippase MurJ, responsible for translocating lipid II across the cytoplasmic membrane, is another well-characterised but clinically unexploited target. MurJ is also referred to as lipid II flippase and is involved in transferring lipid II to the outer cytoplasmic membrane. MurJ is a polytopic inner membrane protein and a member of the multi-drug oligosaccharide-lipid or polysaccharide exporter superfamily [126]. MurJ has now been validated in several GNB and GPB. Inoue A et al. (2008) and Ruiz N (2008) studied the importance of MurJ in cell wall synthesis [127,128]. The studies revealed that MurJ-depleted cells fail to complete the peptidoglycan biosynthesis, leading to the accumulation of precursors and lysis of cells. Kuk et al. (2017) [129], Zheng et al. (2018) [130], and Kohga et al. (2022) [131] proposed the crystal structure of MurJ enzyme. MurJ consists of two homologous six-pass transmembrane bundles, followed by a COOH terminal pair of α-helices located adjacent to the second bundle. The overall inward-open conformation of the protein is mainly because of the presence of two six-helix bundles on the periplasmic face. Although initially controversial, structural data obtained through cryo-electron microscopy and X-ray crystallography suggest that MurJ operates via a rocker-switch mechanism, alternating between inward- and outward-facing conformations to transport lipid II. MurJ is a promising yet untapped target for finding novel antibiotics, motivated by many rationale-driven reasons. It is a pivotal protein involved in peptidoglycan assembly. Moreover, the research efforts of the pharmaceutical industry in the discovery of antibiotics have resulted in the identification of compounds that enhance the activity of β-lactams, restoring their ability to combat bacterial infections. These compounds interact selectively with either MurJ or a homologous protein. Notwithstanding, MurJ inhibition has been reported through the utilization of natural products of the human microbiota and a phage protein. Given these findings, MurJ is a potential target for antibiotics, one that has the capability of significantly improving the efficacy of β-lactams in the clinic. MurJ inhibitors include three classes: (1) humimycins (lipopeptides targeting SAV1754 that restore β-lactam activity against MRSA) [132], (2) phage M lysis protein (from *E. coli* phage M) [133], and (3) small-molecule anthranilic acid/indole derivatives [134]. Their structural diversity (spanning lipopeptides, polypeptides, and small molecules) reveals multiple druggable sites on MurJ, validating its potential as an antibiotic target.

### 3.8. LpxC Enzyme

With a view to tackling MDR infections, another target has been identified; UDP-3O-(*R*-3-hydroxymyristoyl)-*N*-acteylglucosamine deacetylase (LpxC) is a zinc-dependent metalloamidase and has the capacity to catalyse lipid A biosynthesis [135]. LpxC is a single copy gene which is conserved only in GNB. In 2008 Mochalkin et al. reported the crystal structure of LpxC enzyme in *P. aeruginosa*. The structure comprised two α + β domains, each consisting of 30 ligand atoms, two sulfate ions, 6915 protein atoms and 1223 water molecules, five-stranded β sheets and two primary α-helices [136]. The first lead compound, L573,655, against LpxC enzyme of *E. coli* was discovered in the mid-1980s. Subsequent comprehensive studies of LpxC have contributed to the discovery of more potent compounds such as L161,240, CHIR-090, BB-7845, PF 05081090 and ACHN-975. CHIR-090 is a potent lead molecule that inhibits LpxC by chelating the catalytic zinc ion while its hydrophobic moiety occupies the enzyme’s acyl-chain binding tunnel, thereby stabilising the inhibitory complex. However, this antibiotic also developed resistance due to the efflux pump and increased expression of the target, primarily in *P. aeruginosa* [137]. The drug, ACHN-975, is the only LpxC inhibitor known to have been withdrawn from clinical trials due to inflammation at the infusion site.

### 3.9. Auxiliary Cell Wall Enzymes: d,l-Transpeptidases and Lytic Transglycosylases

While D,D-transpeptidases (PBPs) have been classical targets of β-lactam antibiotics, d,l-transpeptidases (Ldts) catalyze alternative cross-linking in certain GPB and GNB. These enzymes generate 3-3 cross-links rather than the 4-3 links formed by PBPs. Ldts are particularly relevant in stationary-phase and dormant cells and are upregulated in certain resistant strains, such as *Mycobacterium tuberculosis* [138].

β-Lactams, except for carbapenems, generally fail to inhibit Ldts, which contributes to drug tolerance and persistence. Selective Ldt inhibitors are still in developmental stages, with limited scaffolds showing efficacy. The potential to combine carbapenems or novel non-β-lactam inhibitors with agents targeting PBPs may enhance efficacy against resistant pathogens [139,140].

Lytic transglycosylases (LTs) cleave glycosidic bonds in the peptidoglycan sacculus, aiding in cell wall remodeling and turnover. Although they are not essential under all growth conditions, their role in pathogenesis, cell division, and adaptation to stress underscores their potential as adjunctive targets [141]. Some LTs also facilitate the incorporation of secretion systems or resistance determinants into the cell wall, linking them to virulence.

### 3.10. Bactoprenol Recycling Enzymes

The lipid carrier bactoprenol (undecaprenyl phosphate) is vital for shuttling peptidoglycan precursors across the membrane. Once its cargo has been unloaded, bactoprenol has to be dephosphorylated and reused to start the transport cycle anew. Enzymes such as UppP/BacA and DedA family proteins, that are involved in this process, have not been thoroughly investigated as drug targets. However, some studies recently have demonstrated that DedA/UptA family proteins can be targeted by lipopeptides, validating their potential as antibacterial targets [142,143].

Inhibition of bactoprenol recycling can lead to depletion of functional lipid carriers, resulting in arrested peptidoglycan synthesis and arrested bacterial growth. Due to their significance and lack in human cells, they are potential untapped targets.

### 3.11. Regulatory Proteins: FtsZ and Divisome Assembly

Cell wall biosynthesis is inevitably linked with cell division and cytoskeletal motion. Temperature-sensitive mutant Z or FtsZ, a tubulin-like GTPase, assembles at the site of upcoming division to create the Z-ring, which is implicated in the recruitment of a set of proteins to create the divisome complex. The complex is implicated in coordinating recruitment and function of PBPs and other cell wall enzymes. It serves as a scaffold to attract divisomes, which are multi-protein complexes, thereby regulating cell wall remodeling and membrane constriction during cytokinesis and additionally it may also produce the force necessary for the bacterial cell survival [144]. FtsZ is also structurally very similar to tubulin, a eukaryotic cytoskeletal protein. While structurally similar, these two proteins are very dissimilar in their structural make-up, GTP binding sites, polymerization characteristics and protein counterparts. They also differ by less than 20% in their amino acid sequences. FtsZ is a 40 kDa molecular weight protein and is coded by the ftsz gene. It also has a GTPase and, in the presence of K^+^ and Mg^2+^, can bind to either guanosine 5′-diphosphate (GDP) or guanosine 5′-triphosphate (GTP) [145]. Depending on its preference for GDP or GTP, FtsZ exists as either higher-order oligomers or as individual monomers. The structure consists of N and C-terminal subglobular domains, each with a unique folding pattern. A central core helix, referred to as H5 and H7, acts to divide the N-terminal domain, connecting it to a C-terminal region with multiple extensions. In each domain is a GTPase-activating site in addition to a nucleotide-binding pocket. In addition, the extended C-terminal tail is important in regulating the activity of many accessory proteins and is essential for the assembly of the FtsZ protofilament [146]. A variety of FtsZ inhibitors, ranging from peptides and natural products to synthetic small molecules, have been identified to interact with different areas of FtsZ. These include the nucleotide-binding pocket in the N-terminal domain, the gap between H7 and the C terminus, and the C terminus itself, ultimately resulting in bacterial cell death. Some of the natural compounds that have been identified to be FtsZ inhibitors include curcumin, cinnamaldehyde, coumarins, totarol, sanguinarine, berberine, viriditoxin, plumbagin, and dichamentin. Some antimicrobial peptides have been identified to be highly potent against FtsZ, including MciZ, CRAMP, Kil peptide, edeines, acyldepsipeptides, FtsZps, NCR247, I19L, N2, and N6 marine peptides, and peptide nucleic acids. Also, a variety of synthetic compounds—such as zantrins, benzamides, benimidazoles, taxanes, arene-diol digallates, pridopyrazine and pyridothiazine analogues, and substituted 1, 6 diphenyl naphthalenes—have also been identified to be highly active against FtsZ [147]. These molecules destabilize FtsZ polymers or block GTP binding/hydrolysis, impairing cytokinesis. Given the central role of FtsZ in cell wall synthesis and its conservation across bacteria, it remains an important yet underexploited target. Other divisome components, such as FtsA, ZipA, FtsW, and FtsI, also play critical roles in assembling and activating cell wall synthesis machinery. Detailed structural and functional studies are needed to validate them as viable drug targets.

### 3.12. Outer Membrane Biogenesis Proteins in GNB

GNB have a second outer membrane, which gives a strong barrier to the majority of antibiotics. Proteins important in its biogenesis—i.e., the Lpt (lipopolysaccharide transport) complex, Bam (β-barrel assembly machinery), and Lol (lipoprotein transport) system—are now increasingly recognized as being crucial to cell envelope integrity.

The β-barrel assembly machinery, or BAM complex, is now recognized as a potential target for antibacterial therapy due to its central role in the assembly of outer membrane proteins (OMPs) in GNB. The OMPs are important in the maintenance of different biological activities in GNB, such as the integrity and stability of the cell shape, and as channels for the transport of proteins. Additionally, OMPs have the ability to act as efflux pumps, a role which is highly correlated with bacterial multidrug resistance, where they can efflux peptides and drugs [148]. Most OMPs are β-barrel proteins, which are barrel structures composed of an even number of antiparallel β-sheets stabilized by lateral hydrogen bonds. There is a long loop on the extracellular side and a short loop on the periplasmic side connecting the β-sheet. In the structure of β-barrel OMPs, the outer surface is hydrophobic and the central region is hydrophilic. BAM plays a crucial role in the assembly of OMPs as it ensures the OMPs fold properly and perform the desired biological processes when inserted into the outer membrane [149]. Some studies have shown that the BAM complex has the ability to increase the folding efficiency of outer membrane proteins (OMPs) and that any interference with the BAM complex has the potential to cause precipitation and abnormal OMP aggregation, which eventually results in bacterial cell death. This indicates that the BAM complex is a promising candidate for the development of new antibiotics. The BAM complex is composed of BamA, a membrane-bound outer membrane protein, and lipoproteins such as BamB, BamC, BamD, and BamE. Of the lipoproteins, BamA, which is highly conserved across GNB, is the core component of the BAM complex, while the other lipoproteins (BamB, BamC, BamD, and BamE) have direct or indirect interactions with BamA, which is attached to the periplasmic surface of the outer membrane [150]. Most of BAM inhibitors (nitazolamide, darobactin, JB-95 and MAB1) reported till date either target BamA or BamD [151,152,153]. Yet, none of these inhibitors have been used therapeutically owing to the fact that there has not been an explicit explanation of the importance of each part of BAM complex and how BAM complex facilitates the folding and insertion of OMPs. LptD and BamA inhibitors have been found to possess strong antibacterial activity, especially against multidrug-resistant GNB. Although these proteins are not directly implicated in peptidoglycan biosynthesis, they play a vital role in the structure and function of the outer membrane, affecting cell wall stability and permeability indirectly. Inhibition of these systems may enhance the effectiveness of conventional cell wall inhibitors by increasing penetration or synergistically disrupting envelope integrity.

### 3.13. Teichoic Acid Biosynthesis

Teichoic acids are the principal anionic polymers of the GPB cell wall, comprising wall teichoic acids (WTAs) and lipoteichoic acids (LTAs). They contribute to cell wall structure, ion homeostasis, and pathogenesis. WTA biosynthesis enzymes, including TarA, TarF/TagF, TarI, TarJ, TarL, TarGH and TarO, have been suggested as antibacterial targets. TarO inhibitors, such as tunicamycin, have been shown to be effective in Staphylococcus aureus models, making bacteria β-lactam susceptible and inhibiting virulence factor expression. But specificity and toxicity are issues, and optimization is needed.

### 3.14. Peptidoglycan O-Acetyltransferases and Amidases

Post-synthetic modifications of peptidoglycan, such as O-acetylation and amidation, influence resistance to host lysozymes and antibiotic susceptibility. Enzymes like O-acetyltransferase A (OatA) and N-acetylmuramoyl-l-alanine amidase are implicated in these processes.

Inhibiting these enzymes could reduce bacterial virulence and augment innate immune clearance. However, few selective inhibitors have been developed, and their structural biology remains to be fully elucidated.

### 3.15. Non-Mevalonate or Methylerythritol 4-phosphate (MEP) Pathway

MEP or non-mevalonate pathway was first reported by Rohmer et al. in 1993 [154]. Isoprenoids are the naturally occurring largest clusters of compounds, having distinct roles in photosynthesis, respiration allelochemical interactions, membrane structure and growth regulation. Most of the organisms synthesize isoprenoids from the 5C precursors, isopentenyl diphopshate and its isomer dimethylallyl diphosphate. The biosynthesis of isoprenoids involves several intermediate enzymes like 1-deoxy-d-xylulose-5-phosphate synthase (Dxs), 1-deoxy-d-xylulose 5-phosphate reductoisomerase (Dxr), 4-diphosphocytidyl-2-C-methyl-d-erythritol synthase (IspD), 4-diphosphocytidyl-2-C-methylerythritol kinase (IspE), 2-C-methyl-d-erythritol 2,4-cyclodiphosphate synthase (IspF), 4-hydroxy-3-methylbut-2-en-1-yl diphosphate synthase (IspG) and 1-hydroxy-2-methyl-butenyl 4-diphosphate reductase (IspH) which have not been investigated extensively. Amongst these, IspG and IspH enzymes were being considered as potential bottlenecks of the MEP pathway. As the MEP pathway is absent from mammals and only confined to major noscomial pathogens, it has been proposed as an appealing target for the development of novel antibacterial agents [155]. Moreover, evidence on possible mechanisms of resistance to block the MEP pathway is scarce. Availability of crystal structures of MEP enzymes pave the way for the discovery of antibacterial agents. The best characterized MEP pathway inhibitor is fosidomycin, first identified as natural wide-spectrum antibiotic targeting Dxr enzyme [156].

### 3.16. Two-Component Regulatory Systems

Bacterial two-component systems (TCS), such as WalKR and VanRS, regulate cell wall synthesis in response to environmental stimuli and antibiotic exposure. These systems modulate the expression of PBPs, autolysins, and stress response genes. Targeting TCS could disrupt cell wall homeostasis and sensitize bacteria to existing antibiotics. WalKR, in particular, is essential in Gram-positive cocci and is being explored as a target for antivirulence strategies. However, the risk of cross-reactivity with eukaryotic kinases must be addressed.

Bacterial histidine kinases (BHK) are promising antibacterial targets and are part of bacterial two-component systems (TCSs), the primary signal transduction pathways that regulate various processes including secretion systems, virulence and antibiotic resistance. TCS signalling involves autophosphorylation of BHK, phosphotransfer of the phosphoryl group to a response regulator, which ultimately modulates the expression of target genes. Autophosphorylation of BHK is mediated via the catalytic and ATP-binding (CA) domain, that binds ATP and phosphorylation of BHK at conserved histidine residue in the dimerization and histidine phosphotransfer (DHp) domain. The CA and DHp domains are conserved in all BHKs, with these conserved features of the BHK CA domain and its role in signal transduction make it an appealing target for phenotypic and structure-based virtual screening of inhibitors [157]. To date, various TCS inhibitors have been described in the literature such as thiazole, bisamidineindole, amidinobenzimidazole, tyramines, 6-oxa isosteres of anacardic acids, salicylanilidines, thienopyridine, hexapeptides, cyanoacetoacetamide, ethodin, phneylocoumarin, DrrA peptide, sulfonamide, diaryltriazolea, thioridazine, carolacton derivatives [158].

The identification and characterization of unexplored targets in bacterial cell wall synthesis expand the possibilities for next-generation antibiotic development. Membrane-associated proteins, regulatory elements, and auxiliary enzymes offer unique advantages such as essentiality, selectivity, and potential to overcome resistance. Advances in genomics, structural biology, and screening platforms are accelerating the discovery and validation of these novel targets. As resistance to classical antibiotics continues to rise, exploiting these lesser-known components may provide the breakthrough needed in the global fight against bacterial infections.

## 4. Structural Insights into Bacterial Cell Wall Targets: Implications for Antibiotic Discovery

Having structural information of the enzymes used in the biosynthesis of bacterial cell walls has greatly helped us to comprehend their catalytic mechanisms, substrate recognition, and conformational dynamics [159]. High-resolution crystal structures of Mur enzymes, PBPs and transglycosylases have been investigated for their antibiotic action and multidrug-resistance, as well as to disclose molecular structures that can be exploited for drug development [160]. Target-specific structural information is highlighted in this review and summarized in Table 1. Examples of such classic structures are the co-crystal structure of PBPs with β-lactam antibiotics that show covalent acylation of the serine active site, and account for the antibacterial activity as well as resistance due to changes in the geometry of the binding [161]. Similarly, structural analysis of MurA in the complex with fosfomycin has clarified the mechanism of alkylation of the catalytic cysteine residue, informing the rational design of fosfomycin analogues [162]. Structural characterisation of Mur ligases and membrane-associated proteins, including MurJ has continued to emphasize the significance of domain motions and conformational changes in catalysis, which are especially pertinent towards addressing dynamic and understudied enzymes [163].

Notably, in a number of emerging and membrane-embedded targets covered in the present review, complete structural information remains limited or fragmentary. However, structures and homology models provide valuable frameworks for detecting conserved motifs, transient pockets, and possible allosteric sites [134]. Structural insights are in this case not a destination but enabling technologies to complement biochemical and genetic investigations, facilitating hypothesis-driven exploration of novel cell wall targets.

In general, structural biology has a supportive but critical role in the development of antibiotics against bacterial cell wall biosynthesis, especially to direct the target prioritization and the discovery of underexploited enzymes in the foreground of this review.

## 5. Inhibitors of Bacterial Cell Wall Synthesis

The inhibition of bacterial cell wall synthesis remains one of the most effective strategies in the treatment of bacterial infections. A diverse array of antibiotics specifically targets key enzymes involved in the biosynthetic pathway of peptidoglycan, a crucial component of the bacterial cell wall. These inhibitors operate by binding to enzymatic active sites, mimicking substrate structures, or covalently modifying catalytic residues, thereby disrupting cell wall construction and leading to bacterial lysis. Table 2 shows a stage-by-stage comparative overview of the clinically relevant and investigational bacterial cell wall synthesis inhibitors that combine data on the timeline of discovery, their mechanism of action, development of resistance and associated compounds. Instead of replicating elaborate descriptions elsewhere in the text, the table is meant to point to larger trends in cytoplasmic, membrane-linked, and extracytoplasmic phases of cell wall assembly.

The major finding made during this comparison is the fact that cytoplasmic inhibitors like fosfomycin and D-cycloserine were found many decades ago with their resistance mechanisms being discovered many years afterward, which highlights how a long-term selective pressure is imposed on them. Conversely, the mechanisms and resistance of membrane-associated and lipid II-targeting compounds are diverse in response to the complexity of the structural and functional complexity of this step. The extracytoplasmic phase is still dominated by β-lactams and analogous classes, in which a huge level of diversification has been urged mostly by resistance evolution. In general, the table can be called a reference framework as it contextualises the trends in historical development and resistance, which do not repeat the mechanistic discussions used in the previous parts.

## 6. Emerging Strategies and Combination Approaches Targeting Cell Wall Biosynthesis

Recent advances in antibacterial development increasingly emphasize not only the discovery of new cell wall-targeting agents but also strategic combination approaches aimed at enhancing efficacy and suppressing resistance.

### 6.1. Therapeutic Combinations and Synergistic Compounds

The development of antibiotic resistance has resulted in the formulation of combination therapies that take advantage of the synergy of interaction among different classes of inhibitors. For example, β-lactams are usually combined with β-lactamase inhibitors or with agents like vancomycin or daptomycin to enhance their effectiveness against resistant bacteria. Other synergistic pairings involve fosfomycin with aminoglycosides or fluoroquinolones, especially in biofilm-associated infections. These pairings augment bacterial killing, limit the emergence of resistance, and increase antibacterial coverage [164].

### 6.2. Emerging and Investigational Inhibitors

High-throughput screening and structure-based drug discovery have resulted in the identification of a number of investigational inhibitors of novel or under-targeted enzymes in the peptidoglycan biosynthesis pathway. These include Mur ligase inhibitors, MurG and MurJ inhibitors, and inhibitors of d,l-transpeptidases and other accessory proteins. Peptidomimetics, nucleoside analogs, and small molecules that utilize new scaffolds are under investigation to overcome resistance and enhance specificity. These inhibitors tend to bind to conserved motifs or allosteric sites and have favorable pharmacokinetic profiles. Fragment-based drug discovery, in conjunction with advanced biophysical techniques, is also yielding promising leads against cell wall synthesis enzymes. Several compounds are in preclinical or early clinical development, holding out the promise of the expansion of the antibacterial armory [165,166,167].

In summary, bacterial cell wall synthesis inhibitors are still a mainstay of antimicrobial chemotherapy. Their multiple mechanisms, structural flexibility, and ability to be combined with other drugs make them highly valuable agents in the treatment of bacterial infections. Additional research and development work must be done to circumvent resistance and extend the application of this critical therapeutic class.

## 7. Author’s Perspective

In light of the rising problem of antimicrobial resistance and the astounding dearth of new antibiotics from clinical pipelines, there is an imperative need to explore new methods to bacterial cell wall biosynthesis. Our research focus addresses the requirement to integrate genomics, proteomics, and structural biology in the discovery of new antibacterial targets of therapeutic value. Such an integrated approach, besides allowing the identification of essential, conserved, and non-redundant enzymes in bacteria, also makes it possible to design mechanism-based inhibitors with minimal off-target effects. Our studies have shown the promise in the exploration of uncharacterized and under-exploited pathways engaged in peptidoglycan synthesis in the later stages. For example, inhibition of enzymes like MurJ and FtsW, central to lipid II precursor translocation, provides a novel opportunity to bypass conventional β-lactam resistance mechanisms. Similarly, enzymes involved in peptidoglycan fragment recycling and autolysin regulation are under-exploited and warrant greater investigation. From a structural and computational point of view, our experiences highlight the importance of structure-guided drug design. Availability of high-resolution structures, coupled with in silico modeling, forms the foundation for rational selection of scaffolds, ligand optimization, and prediction of pharmacokinetic traits. The synergy considerably shortens the preclinical development process. We propose greater utilization of fragment-based screening and molecular dynamics simulations to forecast resistance mutations and plasticity of binding sites, factors greatly influencing efficacy and shelf life of drugs. Furthermore, the combination of natural product libraries, AI-powered molecule generation, and high-throughput screening platforms has the potential to revolutionize the strategies used in the discovery of antibacterial agents. Many studies involve designing hybrid molecules that combine classical β-lactam rings with peptidomimetic backbones to enhance stability and bypass known resistance mechanisms, complemented by machine learning tools that predict bacterial uptake and efflux processes, ensuring only therapeutically active and bioavailable candidates are taken forward for development. Finally, as academic scientists, we believe that collaboration between industry and academia is essential. There is a requirement to build translational activities that translate laboratory discoveries to potential clinical candidates. Policymakers and funding agencies also need to recognize the strategic value of investment in innovative antibiotic development, particularly those against the bacterial cell wall, a key structure for bacterial survival and pathogenicity. Overall, our vision is firmly grounded on the belief that a multidisciplinary approach, combining molecular biology, biophysics, cheminformatics, and clinical pharmacology will provide the next generation of safe, effective, and resistance-resistant antibacterial agents.

## 8. Conclusions

The bacterial cell wall, especially its peptidoglycan layer, is fundamental to bacterial survival and offers a prime target for new antibiotics. While β-lactams have been successful clinically, the rise of multidrug-resistant (MDR) and extensively drug-resistant (XDR) bacteria makes finding new inhibitors and unexplored targets in this pathway critically urgent. Our review emphasizes the intricate regulation of cell wall assembly, spanning precursor synthesis in the cytoplasm, membrane transport, and cross-linking in the periplasm. Although enzymes like PBPs and Mur ligases are well-studied targets, many other structurally distinct and essential proteins remain underutilized. Membrane flippases, autolysins, and peptidoglycan recycling enzymes stand out as particularly promising new targets. Advanced techniques like X-ray crystallography, cryo-EM, structure-based drug design, and computational modelling are now enable the rational design of inhibitors that exploit the dynamic nature of bacterial enzymes. Furthermore, combining natural product screening, synthetic chemistry, and artificial intelligence creates opportunities for developing potent, selective, and bioavailable compounds that can evade resistance. Moving forward, interdisciplinary collaboration is vital to identify and validate novel targets, optimize lead compounds, and convert laboratory findings into effective clinical treatments. With sustained investment and innovation, the bacterial cell wall remains a highly promising source for revolutionary antibiotics, crucial for combating infectious diseases and preserving antimicrobial effectiveness in the future.

## Figures and Tables

**Table 1:** Summary of key targets involved in bacterial cell wall biosynthesis and their representative structural data.

Target	Primary Function	Cellular Location	Representative PDB ID(s)	Pharmacophores/Inhibitors
GlmS	Fructose-6-P → Glucosamine-6-P	Cytoplasmic	1MOQ, 1JXA, 2VF5, 4AMV	6-chloro-5-oxo-l-norleucine, *N*-iodoacetylglucosamine-6-phosphate, 2-amino-2-deoxy-d-glucitol 6-phosphate
GlmM	Phosphoglucosamine mutase	Cytoplasmic	7OJR, 7OML, 7OLH, 7OJS, 6GYZ, 3PDK, 6GYX	2-azido-2-deoxy-α-d-glucopyranosyl phosphate, 2-amino-2,3-dideoxy-3-fluoro-α-*d*-glucopyranosyl phosphate
GlmU	Acetyltransferase/uridyltransferase	Cytoplasmic	1NRK, 2QKX, 3D98, 3D8V, 3DJ4, 3ST8, 4G3Q, 4G87, 6GE9, 1G95, 1G97, 1HM0, 1HM8, 1HM9, 1HV9, 4KPZ, 8CU9	Aminoquinazoline-based molecules
LpxA	Acylation of UDP-GlcNAc	Cytoplasmic	2QIA, 1LXA, 2AQ9, 6P9Q, 5DEM, 5DG3, 7T60, 4MDT, 3PMO	Tetrahydropyran-based derivatives, Pyridopyrimidines/Heterobiaryls, Benzimidazoles, Sulfonyl Piperazines
MurA	Enolpyruvyl transfer to UDP-GlcNAc	Cytoplasmic	1UAE, 3KR6, 3KQJ, 1EJC, 3VCY, 5VM7	Fosfomycin, Avenaciolide, quinazolinone-based derivatives
MurB	Reduction in enolpyruvate moiety	Cytoplasmic	5JZX, 2Q85, 2MBR, 4JAY, 1HSK, 2GQT, 4PYT	Trisubstituted thiazolidinones and imidazolidinone derivatives
MurC	Addition of L-Ala to UDP-MurNAc	Cytoplasmic	2F00, 9D9K, 7BVA, 1P31, 1P3D, 1GQY	Phosphinate transition-state analogues, *N*-acylhydrazones,
MurD	Addition of D-Glu to UDP-MurNAc-L-Ala	Cytoplasmic	1UAG, 2UAG, 1E0D, 1EEH, 2WJP, 5A5F	Peptide-based derivatives, 9H-Xanthene, *N*-Acylhydrazones, ulfonohydrazides, sulfonohydrazones, benzylidenethiazolidinones and pulvinones
MurE	Addition of meso-DAP or L-Lys	Cytoplasmic	1E8C, 2XJA, 8G6P, 4C12, 7D27	Phosphinate derivatives and diaminopimelic acid derivatives
MurF	Addition of D-Ala–D-Ala dipeptide	Cytoplasmic	1GG4, 4QDI, 3ZM6, 8F5D	Aminoalkylphosphinate derivatives
Mpl	Peptide stem recycling	Cytoplasmic	3HN7, 3EAG	Tri, tetra and pentapeptide substrates
D-Amino Acid Aminotransferase (D-AAT)	D-amino acid metabolism	Cytoplasmic	1DAA, 2DAA, 3DAA, 3CSW, 8AHR, 8ONM	D-cycloserine, O-substituted hydroxylamines
MurI (Glutamate racemase)	L-Glu ↔ D-Glu conversion	Cytoplasmic	2JFN, 2DWU, 5HJ7, 5IJW, 2JFQ, 2JFX, 2JFO	l-serine O-sulphate, aziridino glutamate, pyrazolopyrimidinedione
MurG	Lipid I → Lipid II conversion	Cytoplasmic face of inner membrane	1F0K, 1NLM, 7D1I	Uridine Diphosphate (UDP) Mimetics, Muraymycins and Related Liposaccharide Nucleoside Antibiotics
MurJ	Lipid II flippase	Inner membrane	6CC4, 5T77, 6NC9, 7WAG, 6NC6, 6NC7, 6NC8	Humimycins, phage M lysis protein, small-molecule anthranilic acid/indole derivatives
LpxC	Deacetylation in lipid A synthesis	Cytoplasmic	Available 90 PDB structures, Few are 1P42, 2JT2, 4MDT, 7DEM, 8E4A	L161,240, CHIR-090, BB-7845, PF 05081090 and ACHN-975
L,D-Transpeptidases	3–3 peptidoglycan cross-linking	Periplasmic	3DA2, 4ZFQ, 5E5L, 6IYW, 4JMN, 4LZH, 4QTF, 6D4K	Carbapenems, Monobactams, Boronate-Based derivatives, Glycopeptide Derivatives & Lipoglycopeptides, Rhodanine derivatives
Lytic Transglycosylases	Peptidoglycan remodeling	Periplasmic	6GI4, 3T36, 1QUS, 4CFO, 2G6G, 6FC4, 6FCU, 4ANR, 5O24, 6QK4, 8GFP	Bulgecin A and Related Natural Products, Iminosugar-Based Inhibitors, Epoxyalkyl sugars, Arylthiazoles and benzimidazoles, β-Lactam hybrids
Bactoprenol Recycling Enzymes (UppP)	C55PP → C55P	Inner membrane/periplasmic face	6CB2, 4H38, 5OON	Bisphosphonates, Amphomycin and Related Lipopeptides, Aryl-chloroacetamides, Thiazole derivatives, Quinazolinones
Bactoprenol Recycling Enzymes (PgpB)	C55PP → C55P	Inner membrane	4PX7, 5JWY	Vanadate, Diacylglycerol Pyrophosphate (DGPP) Analogues, Bisphosphonates.
FtsZ	Z-ring formation (divisome)	Cytoplasmic	6UMK, 6UNX, 6LL6, 3VOB, 3VO8, 5H5G, 5XDT, 2QIY, 6YM1, 1FSZ	Antimicrobial peptides, zantrins, benzamides, benimidazoles, taxanes, arene-diol digallates, pridopyrazine and pyridothiazine analogues, and substituted 1, 6 diphenyl naphthalene derivatives
Divisome proteins (FtsA)	Septal ring stabilization	Cytoplasmic	4A2A, 1E4F, 4A2B, 7Q6D, 7Q6F, 7Q61, 3WQU	
Outer membrane biogenesis (BamA)	OM protein assembly (GNB)	Outer membrane	4K3B, 4N75, 5D0Q, 5LJO, 8BWC, 7NRF, 7NRE, 7R1W, 7TT7	Nitazolamide, darobactin, JB-95 and MAB1
Teichoic acid biosynthesis (TarA, TarF/TagF, TarI, TarJ, TarL, TarGH and TarO)	Initiation of WTA synthesis	Cytoplasmic	5WFG, 3L7L, 3L71, 3L7J, 7QXC and 6JBH	Tunicamycin
Peptidoglycan O-acetyltransferase (OatA)	O-acetylation of PG	Membrane/periplasmic	6WN9, 6VJP, 4JHL, 2VPT, 5B5S, 5UFY, 8TLB, 7TRR	Chloroacetamide derivatives, Anacardic acid, curcumin, Aryl sulfonamides, benzimidazoles, CoA analogues, pantetheine derivatives
N-acetylmuramoyl-L-alanine amidase	Cell separation/remodeling	Periplasmic	8C4D, 5EMI, 7AGO, 3D2Z, 4BIN, 6SSC	Succinylhydroxamate, DTT, Hydroxamic acids, Thiols, Benzimidazoles, Cationic peptides, Isoxazolidinones,
MEP pathway enzymes (IspG and IspH)	Isoprenoid precursor synthesis	Cytoplasmic	3NOY, 4G9P, 4S3F, 3F7T, 3KE8, 3ZGL, 4MUX, 3DNF, 1WHJ	fosidomycin, Bisphosphonates, Cyclodiphosphate Analogues, Triazole and Tetrazole Derivatives, Cyanide and Azide Derivatives, Phenothiazines
Two-component systems (Histidine kinase domains)	Cell wall stress sensing	Membrane/cytoplasmic	3DGE, 1EAY, 6LGQ,	Thiazole, bisamidineindole, amidinobenzimidazole, tyramines, 6-oxa isosteres of ana-cardic acids, salicylanilidines, thienopyridine, hexapeptides, cyanoacetoacetamide, ethodin, phneylocoumarin, DrrA peptide, sulfonamide, diaryltriazolea, thioridazine, carolacton derivatives

**Table 2:** Brief outline of cell wall synthesis inhibitors.

Stages of Cell Wall Synthesis	Drug	Discovered/FDA Approved Year	Source	MOA	Resistance	Resistance Discovered Year	Other Drugs (Year of Discovery)
Cytoplasmic stage	Fosfomycin	1969	*Pseudomonas syringae* and *Streptomyces* sp.	Inhibition of MurA by forming a thioether bond with Cys117	Mutations in MurA and the synthesis of Fosfomycin inactivating proteins (FosA)	2000 & 2010	Not yet discovered
	D-cycloserine	1954	*Streptomyces* sp.	Inhibition of d-Ala-d-Ala ligase (Ddl), d-Ala racemase (Alr)	Upregulation of Alr gene in *M. smegmatis* and also point mutations in CycA gene	1965	O-carbamyl-d-serine, Chlorvinyl glycine, alafosfalin
	Vancomycin	1954	*Amycolatopsis orientalis*	d-Ala-d-Ala terminal of peptide backbone of murein precursor, lipid II	Transfer of resistant genes and impermeability of cell wall	1988	Avoparcin (used in feed stock), ristocetin (discontinued), complestatin, corbomycin, teicoplanin, telavancin (2009), dalbavancin (2014) and oritavancin (2014)
Membrane associated stage	Nucleoside antibiotics	1971	-	Inhibition of MraY	Mutations and efflux pumps	-	Tunicamycin (1971), lipsidomycins (1985), mureidomycins (1989), pacidamycins, and capuramycins
	ramoplanin	Phase III (VREF) and Phase II (MRSA)	*Actinoplanes*	Inhibition of MurG enzyme or sequestering of Lipid II	Cross resistance to other drugs	-	Janiemycin
	Nisin	1928	*Lactococcus lactis*	Pore formation in Lipid II	Drug inactivation, ABC transporters and cell wall modification	1952	Mersacidin, Teixobactin
	Lantibiotics (NVB302)	1990 (discontinued in the year 2018)	semisynthetic	Binds to lipid II	Not reported	-	Derivatives of NVB302 (under development)
Extra cytoplasmic stage	Penicillin	1940	*Penicillium notatum*	Binds to PBPs	enzymatic degradation by the overexpressed β-lactamases	1967	Penicillin G, Penicillin V, Methicillin, Cloxacillin, Nafcillin, Oxacillin, dicloxacillin, Ampicillin, Amoxicillin and Hetacillin, Carbenicillin, Ticarcillin, piperacillin, Azlocillin, Mezlocillin, amidinocillin, sulbenicillin, Clavulanic acid, sulbactam
	Cephalosporins	1945, 1948	*Acremonium*	Binds to PBPs	CTX-M-type-ESBLs and AmpC β-lactamases	1983	Cefazolin, Cephalexin, Cefadroxil, Cefapirin, Cefazedone, Ceftezole, Cefazaflur, Cefalonium, Cafaloridine, Cefatrizine, Cefaloglycin, Cefadrine, Cefalotin, Cefroxadine and Cefacetrile, Cefotetan, Cefoxitin, Cefuroxime, Cefaclor, Cefotetan, Cefoxitin, Cefprozil, Cefamandole, Cefminox, Cefonicid, Ceforanide, Cefbuperazone, Cefuzonam, Cefmetazole, Carbacephem, Loracarbef, Cefotaxime, Ceftazidime, Ceftriaxone, Cefdinir, Cefixime, Cefoperazone, Cefcapene, Cefdaloxime, Ceftizoxime, Cefmenoxime, Cefpiramide, Cefpodoxime, Ceftibuten, Cefditoren, Cefotiam, Cefetamet, Cefodizime, Cefpimizole, Cefsulodin, Cefteram, Ceftiolene, Oxacephem, Cefepime, Cefozopran, Cefpirome, Cefquinome, Ceftaroline, Ceftolozane, Ceftobiprole
	Carbapenems	1976	*Streptomyces olivaceus*	Bind to PBPs	Alteration of porin channels, reduced membrane permeability to β-lactams and production of carbapenemases	1991	Olivanic acids, Impenem (1985), meropenem (1996), ertapenem, doripenem, panipenem (JA-1993), biapenem (JA-2001), tebipenem (JA-2015), razupenem, lenapenem, tomopenem, thienamycin (1976)
	Monobactams	1981	*Chromobacterium* sp.	Inhibits peptidoglycan cross linking and also bind to PBPs	Alteration of PBPs, decreased permeability	-	Aztreonam, sulbactam, clavulanic acid, tigemonam, nocardicin A, carumonam and tabtoxin
	Siderophore conjugates	1947	-	Binds to PBPs	Loss of catecholate receptors	-	Albomycin (1947), Cefiderocol (2019) GSK-2696266 and BAL30072
	Moenomycin	1960 (failed in developmental trials in humans)	*Streptomyces ghanaensis*	Inhibits peptidoglycan glucosyltransferases	Mutations of glucosyltransferases	-	Flavomycin, Katanosin and plubascin A3
	Bacitracin	1943	*Bacillus subtilis* and *Bacillus licheniformis*	Forms a complex with undecaisoprenyl pyrophosphate, thereby preventing the formation of phosphate which is carrier for the peptidoglycan building blocks	Mutations in genes bcrA and bcrB causing resistance in *Clostridium perfringens* strain	-	-
	L573,655 (lead molecule)	Mid-1980s	semisynthetic	LpxC inhibition	-	-	L161,240, CHIR-090, BB-7845, PF 05081090, ACHN-975 (clinical trials)

## Data Availability

No new data were created.

## Abbreviations

The following abbreviations are used in this manuscript:

MDR	Multidrug-resistant
XDR	Extensively drug-resistant
Ddl	d-Ala ligase
Alr	d-Ala racemase
cryo-EM	Cryo-electron microscopy
NMR	Nuclear magnetic resonance
FBDD	Fragment-based drug discovery
SBDD	Structure-based drug design
PBPs	Penicillin-binding proteins
DHp	Dimerization and histidine phosphotransfer
CA	Catalytic and ATP-binding domain
TCS	Two-component systems
BHK	Bacterial histidine kinases
IspH	1-hydroxy-2-methyl-butenyl 4-diphosphate reductase
IspG	4-hydroxy-3-methylbut-2-en-1-yl diphosphate synthase
IspF	2-C-methyl-d-erythritol 2,4-cyclodiphosphate synthase
IspE	4-diphosphocytidyl-2-C-methylerythritol kinase
IspD	4-diphosphocytidyl-2-C-methyl-d-erythritol synthase
Dxr	1-deoxy-d-xylulose 5-phosphate reductoisomerase
Dxs	1-deoxy-d-xylulose-5-phosphate synthase
MEP	Non-mevalonate or methylerythritol 4-phosphate pathway
OatA	O-acetyltransferase A
LTAs	Lipoteichoic acids
WTAs	Wall teichoic acids
OMPs	Outer membrane proteins
PBPs	D,D-transpeptidases
LpxC	UDP-3O-(*R*-3-hydroxymyristoyl)-*N*-acteylglucosamine deacetylase
d-AAT	*d*-Amino acid aminotransferase
Mpl	Murein peptide ligase
PG	Peptidoglycan
mA_2_pm	*meso*-diaminopimelic acid

## References

[1] Salam M.A., Al-Amin M.Y., Salam M.T., Pawar J.S., Akhter N., Rabaan A.A., Alqumber M.A. (2023). Antimicrobial resistance: A growing serious threat for global public health. Healthcare.

[2] Ferri M., Ranucci E., Romagnoli P., Giaccone V. (2017). Antimicrobial resistance: A global emerging threat to public health systems. Crit. Rev. Food Sci. Nutr..

[3] World Health Organization (2024). Global Research Agenda for Antimicrobial Resistance in Human Health.

[4] Hauser A. (2018). Antibiotic Basics for Clinicians.

[5] Walsh C. (2003). Antibiotics: Actions, Origins, Resistance.

[6] Scheffers D.-J., Pinho M.G. (2005). Bacterial cell wall synthesis: New insights from localization studies. Microbiol. Mol. Biol. Rev..

[7] Liu Y., Breukink E. (2016). The membrane steps of bacterial cell wall synthesis as antibiotic targets. Antibiotics.

[8] Barreteau H., Kovač A., Boniface A., Sova M., Gobec S., Blanot D. (2008). Cytoplasmic steps of peptidoglycan biosynthesis. FEMS Microbiol. Rev..

[9] de Kruijff B., van Dam V., Breukink E. (2008). Lipid II: A central component in bacterial cell wall synthesis and a target for antibiotics. Prostaglandins Leukot. Essent. Fat. Acids.

[10] Walter A., Mayer C. (2019). Peptidoglycan structure, biosynthesis, and dynamics during bacterial growth. Extracellular Sugar-Based Biopolymers Matrices.

[11] Sarkar P., Haldar J. (2019). Glycopeptide antibiotics: Mechanism of action and recent developments. Antibiotic Drug Resistance.

[12] Binda E., Marinelli F., Marcone G.L. (2014). Old and new glycopeptide antibiotics: Action and resistance. Antibiotics.

[13] Yoo J., Mashalidis E.H., Kuk A.C., Yamamoto K., Kaeser B., Ichikawa S., Lee S.-Y. (2018). GlcNAc-1-P-transferase–tunicamycin complex structure reveals basis for inhibition of N-glycosylation. Nat. Struct. Mol. Biol..

[14] Kernodle D.S. (2006). Mechanisms of Resistance to β-Lactam Antibiotics. Gram-Positive Pathogens.

[15] Kumar S., Mollo A., Kahne D., Ruiz N. (2022). The bacterial cell wall: From lipid II flipping to polymerization. Chem. Rev..

[16] Ng V., Chan W.C. (2016). New found hope for antibiotic discovery: Lipid II inhibitors. Chem.–A Eur. J..

[17] Ling L.L., Schneider T., Peoples A.J., Spoering A.L., Engels I., Conlon B.P., Mueller A., Schäberle T.F., Hughes D.E., Epstein S. (2015). A new antibiotic kills pathogens without detectable resistance. Nature.

[18] Garde S., Chodisetti P.K., Reddy M. (2021). Peptidoglycan: Structure, synthesis, and regulation. EcoSal Plus.

[19] Torrens G., Cava F. (2024). Mechanisms conferring bacterial cell wall variability and adaptivity. Biochem. Soc. Trans..

[20] Bagdad Y., Miteva M.A. (2024). Recent applications of artificial intelligence in discovery of new antibacterial agents. Adv. Appl. Bioinform. Chem..

[21] Gangwal A., Lavecchia A. (2025). Artificial Intelligence in Natural Product Drug Discovery: Current Applications and Future Perspectives. J. Med. Chem..

[22] Vila J., Moreno-Morales J., Ballesté-Delpierre C. (2020). Current landscape in the discovery of novel antibacterial agents. Clin. Microbiol. Infect..

[23] Monaghan R.L., Barrett J.F. (2006). Antibacterial drug discovery—Then, now and the genomics future. Biochem. Pharmacol..

[24] Sauvage E., Terrak M. (2016). Glycosyltransferases and transpeptidases/penicillin-binding proteins: Valuable targets for new antibacterials. Antibiotics.

[25] Miyachiro M.M., Contreras-Martel C., Dessen A. (2019). Penicillin-binding proteins (PBPs) and bacterial cell wall elongation complexes. Macromol. Protein Complexes II Struct. Funct..

[26] Gloster T.M. (2014). Advances in understanding glycosyltransferases from a structural perspective. Curr. Opin. Struct. Biol..

[27] Halliday J., McKeveney D., Muldoon C., Rajaratnam P., Meutermans W. (2006). Targeting the forgotten transglycosylases. Biochem. Pharmacol..

[28] Batson S., de Chiara C., Majce V., Lloyd A.J., Gobec S., Rea D., Fülöp V., Thoroughgood C.W., Simmons K.J., Dowson C.G. (2017). Inhibition of D-Ala: D-Ala ligase through a phosphorylated form of the antibiotic D-cycloserine. Nat. Commun..

[29] Zhao H., Sun Y., Peters J.M., Gross C.A., Garner E.C., Helmann J.D. (2016). Depletion of undecaprenyl pyrophosphate phosphatases disrupts cell envelope biogenesis in Bacillus subtilis. J. Bacteriol..

[30] Oluwole A.O., Hernández-Rocamora V.M., Cao Y., Li X., Vollmer W., Robinson C.V., Bolla J.R. (2024). Real-time biosynthetic reaction monitoring informs the mechanism of action of antibiotics. J. Am. Chem. Soc..

[31] Cudic M., Fields G.B. (2009). Extracellular proteases as targets for drug development. Curr. Protein Pept. Sci..

[32] Maddocks S.E. (2016). Novel targets of antimicrobial therapies. Microbiol. Spectr..

[33] Peraman R., Sure S.K., Dusthackeer V.A., Chilamakuru N.B., Yiragamreddy P.R., Pokuri C., Kutagulla V.K., Chinni S. (2021). Insights on recent approaches in drug discovery strategies and untapped drug targets against drug resistance. Future J. Pharm. Sci..

[34] Ghosh S., Blumenthal H.J., Davidson E., Roseman S. (1960). Glucosamine metabolism: V. enzymatic synthesis of glucosamine 6-phosphate. J. Biol. Chem..

[35] Badet-Denisot M.-A., Rene L., Badet B. (1993). Mechanistic investigations on glucosamine-6-phosphate synthase. Bull. De La Société Chim. De Fr..

[36] Massière F., Badet-Denisot M.-A. (1998). The mechanism of glutamine-dependent amidotransferases. Cell. Mol. Life Sci. CMLS.

[37] Kort M. (1970). Reactions of free sugars with aqueous ammonia. Advances in Carbohydrate Chemistry and Biochemistry.

[38] Golinelli-Pimpaneau B.a., Le Goffic F., Badet B. (1989). Glucosamine-6-phosphate synthase from *Escherichia coli*: Mechanism of the reaction at the fructose 6-phosphate binding site. J. Am. Chem. Soc..

[39] Mei B., Zalkin H. (1990). Amino-terminal deletions define a glutamine amide transfer domain in glutamine phosphoribosylpyrophosphate amidotransferase and other PurF-type amidotransferases. J. Bacteriol..

[40] Zalkin H. (1993). The amidotransferases. Advances in Enzymology and Related Areas of Molecular Biology.

[41] Denisot M.-A., Le Goffic F., Badet B. (1991). Glucosamine-6-phosphate synthase from *Escherichia coli* yields two proteins upon limited proteolysis: Identification of the glutamine amidohydrolase and 2R ketose/aldose isomerase-bearing domains based on their biochemical properties. Arch. Biochem. Biophys..

[42] Corizzi V., Badet B., Badet-Denisot M.-A. (1992). Stereoselective synthesis of the 6-phosphono analogue of fructose-6-phosphate. J. Chem. Soc. Chem. Commun..

[43] Badet-Denisot M.-A., Leriche C., Massière F., Badet B. (1995). Nitrogen transfer in *E. coli* glucosamine-6P synthase. Investigations using substrate and bisubstrate analogs. Bioorganic Med. Chem. Lett..

[44] Bearne S.L. (1996). Active site-directed inactivation of *Escherichia coli* glucosamine-6-phosphate synthase: Determination of the fructose 6-phosphate binding constant using a carbohydrate-based inactivator. J. Biol. Chem..

[45] Leriche C., Badet-Denisot M.A., Badet B. (1997). Affinity Labeling of *Escherichia coli* Glucosamine-6-Phosphate Synthase with a Fructose 6-Phosphate Analog: Evidence for Proximity Between the N-Terminal Cysteine and the Fructose-6-Phosphate-Binding Site. Eur. J. Biochem..

[46] Buchanan J.M. (1973). The amidotransferases. Advances in Enzymology and Related Areas of Molecular Biology.

[47] Bearne S.L., Blouin C. (2000). Inhibition of *Escherichia coli* Glucosamine-6-phosphate Synthase by Reactive Intermediate Analogues: THE ROLE OF THE 2-AMINO FUNCTION IN CATALYSIS. J. Biol. Chem..

[48] Mehra-Chaudhary R., Mick J., Beamer L.J. (2011). Crystal structure of *Bacillus anthracis* phosphoglucosamine mutase, an enzyme in the peptidoglycan biosynthetic pathway. J. Bacteriol..

[49] Regni C., Naught L., Tipton P.A., Beamer L.J. (2004). Structural basis of diverse substrate recognition by the enzyme PMM/PGM from P. aeruginosa. Structure.

[50] Neidhardt F.C. (1996). Escherichia coli and Salmonella: Cellular and Molecular Biology.

[51] Raetz C.R. (1986). Molecular genetics of membrane phospholipid synthesis. Annu. Rev. Genet..

[52] De Reuse H., Labigne A., Mengin-Lecreulx D. (1997). The Helicobacter pylori ureC gene codes for a phosphoglucosamine mutase. J. Bacteriol..

[53] Jolly L., Wu S., van Heijenoort J., de Lencastre H., Mengin-Lecreulx D., Tomasz A. (1997). The femR315 gene from *Staphylococcus aureus*, the interruption of which results in reduced methicillin resistance, encodes a phosphoglucosamine mutase. J. Bacteriol..

[54] Tavares I.M., Jolly L., Pompeo F., Leitão J.H., Fialho A.N.M., Sá-Correia I., Mengin-Lecreulx D. (2000). Identification of the *Pseudomonas aeruginosa glmM* gene, encoding phosphoglucosamine mutase. J. Bacteriol..

[55] Shimazu K., Takahashi Y., Uchikawa Y., Shimazu Y., Yajima A., Takashima E., Aoba T., Konishi K. (2008). Identification of the Streptococcus gordonii glmM gene encoding phosphoglucosamine mutase and its role in bacterial cell morphology, biofilm formation, and sensitivity to antibiotics. FEMS Immunol. Med. Microbiol..

[56] Dai J., Liu Y., Ray W., Konno M. (1992). The crystal structure of muscle phosphoglucomutase refined at 2.7-angstrom resolution. J. Biol. Chem..

[57] Li Y., Zhou Y., Ma Y., Li X. (2011). Design and synthesis of novel cell wall inhibitors of *Mycobacterium tuberculosis* GlmM and GlmU. Carbohydr. Res..

[58] Blaukat A., Abd Alla S., Lohse M.J., Müller-Esterl W. (1996). Ligand-induced phosphorylation/dephosphorylation of the endogenous bradykinin B2 receptor from human fibroblasts. J. Biol. Chem..

[59] Kang J., Xu L., Yang S., Yu W., Liu S., Xin Y., Ma Y. (2013). Effect of phosphoglucosamine mutase on biofilm formation and antimicrobial susceptibilities in *M. smegmatis glmM* gene knockdown strain. PLoS ONE.

[60] Gehring A.M., Lees W.J., Mindiola D.J., Walsh C.T., Brown E.D. (1996). Acetyltransfer precedes uridylyltransfer in the formation of UDP-N-acetylglucosamine in separable active sites of the bifunctional GlmU protein of *Escherichia coli*. Biochemistry.

[61] Brown K., Pompeo F., Dixon S., Mengin-Lecreulx D., Cambillau C., Bourne Y. (1999). Crystal structure of the bifunctional N-acetylglucosamine 1-phosphate uridyltransferase from *Escherichia coli*: A paradigm for the related pyrophosphorylase superfamily. EMBO J..

[62] Tran A.T., Wen D., West N.P., Baker E.N., Britton W.J., Payne R.J. (2013). Inhibition studies on *Mycobacterium tuberculosis* N-acetylglucosamine-1-phosphate uridyltransferase (GlmU). Org. Biomol. Chem..

[63] Soni V., Suryadevara P., Sriram D., Kumar S., Nandicoori V.K., Yogeeswari P. (2015). Structure-based design of diverse inhibitors of *Mycobacterium tuberculosis* N-acetylglucosamine-1-phosphate uridyltransferase: Combined molecular docking, dynamic simulation, and biological activity. J. Mol. Model..

[64] Mehra R., Rani C., Mahajan P., Vishwakarma R.A., Khan I.A., Nargotra A. (2016). Computationally guided identification of novel *Mycobacterium tuberculosis* GlmU inhibitory leads, their optimization, and in vitro validation. ACS Comb. Sci..

[65] Rani C., Mehra R., Sharma R., Chib R., Wazir P., Nargotra A., Khan I.A. (2015). High-throughput screen identifies small molecule inhibitors targeting acetyltransferase activity of *Mycobacterium tuberculosis* GlmU. Tuberculosis.

[66] Kouidmi I., Levesque R.C., Paradis-Bleau C. (2014). The biology of Mur ligases as an antibacterial target. Mol. Microbiol..

[67] Hervin V., Roy V., Agrofoglio L.A. (2023). Antibiotics and Antibiotic Resistance—Mur Ligases as an Antibacterial Target. Molecules.

[68] Skarzynski T., Mistry A., Wonacott A., Hutchinson S.E., Kelly V.A., Duncan K. (1996). Structure of UDP-N-acetylglucosamine enolpyruvyl transferase, an enzyme essential for the synthesis of bacterial peptidoglycan, complexed with substrate UDP-N-acetylglucosamine and the drug fosfomycin. Structure.

[69] Aboushady Y. (2023). Design and Synthesis of MurA Enzyme Inhibitors and Their Evaluation as Antibacterial Agents. Ph.D. Thesis.

[70] Chang C.-M., Chern J., Chen M.-Y., Huang K.-F., Chen C.-H., Yang Y.-L., Wu S.-H. (2015). Avenaciolides: Potential MurA-targeted inhibitors against peptidoglycan biosynthesis in methicillin-resistant *Staphylococcus aureus* (MRSA). J. Am. Chem. Soc..

[71] Benson T.E., Marquardt J.L., Marquardt A.C., Etzkorn F.A., Walsh C.T. (1993). Overexpression, purification, and mechanistic study of UDP-N-acetylenolpyruvylglucosamine reductase. Biochemistry.

[72] Benson T.E., Walsh C.T., Hogle J.M. (1997). X-ray crystal structures of the S229A mutant and wild-type MurB in the presence of the substrate enolpyruvyl-UDP-N-acetylglucosamine at 1.8-Å resolution. Biochemistry.

[73] Benson T.E., Walsh C.T., Massey V. (1997). Kinetic characterization of wild-type and S229A mutant MurB: Evidence for the role of Ser 229 as a general acid. Biochemistry.

[74] Benson T.E., Harris M.S., Choi G.H., Cialdella J.I., Herberg J.T., Martin J.P., Baldwin E.T. (2001). A structural variation for MurB: X-ray crystal structure of *Staphylococcus* aureus UDP-N-acetylenolpyruvylglucosamine reductase (MurB). Biochemistry.

[75] Sylvester D.R., Alvarez E., Patel A., Ratnam K., Kallender H., Wallis N.G. (2001). Identification and characterization of UDP-N-acetylenolpyruvylglucosamine reductase (MurB) from the gram-positive pathogen *Streptococcus pneumoniae*. Biochem. J..

[76] Katz A.H., Caufield C.E. (2003). Structure-based design approaches to cell wall biosynthesis inhibitors. Curr. Pharm. Des..

[77] Andres C.J., Bronson J.J., D’Andrea S.V., Deshpande M.S., Falk P.J., Grant-Young K.A., Harte W.E., Ho H.-T., Misco P.F., Robertson J.G. (2000). 4-Thiazolidinones: Novel inhibitors of the bacterial enzyme MurB. Bioorganic Med. Chem. Lett..

[78] Bronson J.J., DenBleyker K.L., Falk P.J., Mate R.A., Ho H.-T., Pucci M.J., Snyder L.B. (2003). Discovery of the first antibacterial small molecule inhibitors of MurB. Bioorganic Med. Chem. Lett..

[79] Reck F., Marmor S., Fisher S., Wuonola M.A. (2001). Inhibitors of the bacterial cell wall biosynthesis enzyme MurC. Bioorganic Med. Chem. Lett..

[80] Liger D., Masson A., Blanot D., Van Heijenoort J., Parquet C. (1995). Over-production, Purification and Properties of the Uridine-diphosphate-N-Acetylmuramate: L-alanine Ligase from *Escherichia coli*. Eur. J. Biochem..

[81] Mahapatra S., Crick D.C., Brennan P.J. (2000). Comparison of the UDP-N-acetylmuramate: L-alanine ligase enzymes from *Mycobacterium tuberculosis* and *Mycobacterium leprae*. J. Bacteriol..

[82] El Zoeiby A., Sanschagrin F., Lamoureux J., Darveau A., Levesque R.C. (2000). Cloning, over-expression and purification of *Pseudomonas aeruginosa* murC encoding uridine diphosphate N-acetylmuramate: L-alanine ligase. FEMS Microbiol. Lett..

[83] Hesse L., Bostock J., Dementin S., Blanot D., Mengin-Lecreulx D., Chopra I. (2003). Functional and biochemical analysis of *Chlamydia trachomatis* MurC, an enzyme displaying UDP-N-acetylmuramate: Amino acid ligase activity. J. Bacteriol..

[84] Emanuele J.J., Jin H., Yanchunas J., Villafranca J.J. (1997). Evaluation of the kinetic mechanism of *Escherichia coli* uridine diphosphate-N-acetylmuramate: L-alanine ligase. Biochemistry.

[85] Mol C.D., Brooun A., Dougan D.R., Hilgers M.T., Tari L.W., Wijnands R.A., Knuth M.W., McRee D.E., Swanson R.V. (2003). Crystal structures of active fully assembled substrate-and product-bound complexes of UDP-N-acetylmuramic acid: L-alanine ligase (MurC) from *Haemophilus influenzae*. J. Bacteriol..

[86] Fiuza M., Canova M.J., Patin D., Letek M., Zanella-Cléon I., Becchi M., Mateos L.M., Mengin-Lecreulx D., Molle V., Gil J.A. (2008). The MurC ligase essential for peptidoglycan biosynthesis is regulated by the serine/threonine protein kinase PknA in *Corynebacterium glutamicum*. J. Biol. Chem..

[87] Gordon E., Flouret B., Chantalat L., van Heijenoort J., Mengin-Lecreulx D., Dideberg O. (2001). Crystal structure of UDP-N-acetylmuramoyl-L-alanyl-D-glutamate: Meso-diaminopimelate ligase from *Escherichia coli*. J. Biol. Chem..

[88] Bertrand J.A., Auger G., Fanchon E., Martin L., Blanot D., Van Heijenoort J., Dideberg O. (1997). Crystal structure of UDP-N-acetylmuramoyl-L-alanine: D-glutamate ligase from *Escherichia coli*. EMBO J..

[89] Emanuele J.J., Jin H., Jacobson B.L., Chang C.Y., Einspahr H.M., Villafranca J.J. (1996). Kinetic and crystallographic studies of *Escherichia coli* UDP-N-acetylmuramate: L-alanine ligase. Protein Sci..

[90] Šink R., Kovač A., Tomašić T., Rupnik V., Boniface A., Bostock J., Chopra I., Blanot D., Mašič L.P., Gobec S. (2008). Synthesis and biological evaluation of N-acylhydrazones as inhibitors of MurC and MurD ligases. ChemMedChem Chem. Enabling Drug Discov..

[91] Schleifer K.H., Kandler O. (1972). Peptidoglycan types of bacterial cell walls and their taxonomic implications. Bacteriol. Rev..

[92] Auger G., Martin L., Bertrand J., Ferrari P., Fanchon E., Vaganay S., Pétillot Y., van Heijenoort J., Blanot D., Dideberg O. (1998). Large-Scale Preparation, Purification, and Crystallization of UDP-N-Acetylmuramoyl-l-Alanine: D-Glutamate Ligase from *Escherichia coli*. Protein Expr. Purif..

[93] Walsh A.W., Falk P.J., Thanassi J., Discotto L., Pucci M.J., Ho H.-T. (1999). Comparison of the D-glutamate-adding enzymes from selected gram-positive and gram-negative bacteria. J. Bacteriol..

[94] Pratviel-Sosa F., Mengin-Lecreulx D., van Heijenoort J. (1991). Over-production, purification and properties of the uridine diphosphate N-acetylmuramoyl-l-alanine: D-glutamate ligase from *Escherichia coli*. Eur. J. Biochem..

[95] Bertrand J.A., Auger G., Martin L., Fanchon E., Blanot D., Le Beller D., van Heijenoort J., Dideberg O. (1999). Determination of the MurD mechanism through crystallographic analysis of enzyme complexes. J. Mol. Biol..

[96] Kotnik M., Humljan J., Contreras-Martel C., Oblak M., Kristan K., Hervé M., Blanot D., Urleb U., Gobec S., Dessen A. (2007). Structural and functional characterization of enantiomeric glutamic acid derivatives as potential transition state analogue inhibitors of MurD ligase. J. Mol. Biol..

[97] Todhunter J.A., Purich D.L. (1975). Use of the sodium borohydride reduction technique to identify a gamma-glutamyl phosphate intermediary in the *Escherichia coli* glutamine synthetase reaction. J. Biol. Chem..

[98] Gupta A., Pal S.K., Pandey D., Fakir N.A., Rathod S., Sinha D., SivaKumar S., Sinha P., Periera M., Balgam S. (2017). PknB remains an essential and a conserved target for drug development in susceptible and MDR strains of M. Tuberculosis. Ann. Clin. Microbiol. Antimicrob..

[99] Joshi A.A., Narkhede S.S., Viswanathan C. (2005). Design, synthesis and evaluation of 5-substituted amino-2, 4-diamino-8-chloropyrimido-[4,5-b] quinolines as novel antimalarials. Bioorganic Med. Chem. Lett..

[100] Frlan R., Kovač A., Blanot D., Gobec S., Pečar S., Obreza A. (2011). Design, Synthesis and in vitro Biochemical Activity of Novel Amino Acid Sulfonohydrazide Inhibitors of MurC. Acta Chim. Slov..

[101] Frlan R., Kovač A., Blanot D., Gobec S., Pečar S., Obreza A. (2008). Design and synthesis of novel N-benzylidenesulfonohydrazide inhibitors of MurC and MurD as potential antibacterial agents. Molecules.

[102] Tomašić T., Zidar N., Kovač A., Turk S., Simčič M., Blanot D., Müller-Premru M., Filipič M., Grdadolnik S.G., Zega A. (2010). 5-Benzylidenethiazolidin-4-ones as multitarget inhibitors of bacterial Mur ligases. ChemMedChem Chem. Enabling Drug Discov..

[103] Antane S., Caufield C.E., Hu W., Keeney D., Labthavikul P., Naughton S.M., Petersen P.J., Rasmussen B.A., Singh G., Yang Y. (2006). Pulvinones as bacterial cell wall biosynthesis inhibitors. Bioorganic Med. Chem. Lett..

[104] Mengin-Lecreulx D., Falla T., Blanot D., Van Heijenoort J., Adams D.J., Chopra I. (1999). Expression of the *Staphylococcus aureus* UDP-N-acetylmuramoyl-L-alanyl-D-glutamate: L-lysine ligase in *Escherichia coli* and effects on peptidoglycan biosynthesis and cell growth. J. Bacteriol..

[105] Mengin-Lecreulx D., Michaud C., Richaud C., Blanot D., Van Heijenoort J. (1988). Incorporation of LL-diaminopimelic acid into peptidoglycan of *Escherichia coli* mutants lacking diaminopimelate epimerase encoded by dapF. J. Bacteriol..

[106] Michaud C., Mengin-Lecreulx D., van Heijenoort J., Blanot D. (1990). Over-production, purification and properties of the uridine-diphosphate-N-acetylmuramoyl-l-alanyl-d-glutamate: Meso-2, 6-diaminopimelate ligase from *Escherichia coli*. Eur. J. Biochem..

[107] Hammes W.P., Neukam R., Kandler O. (1977). On the specificity of the uridine diphospho-N-acetylmuramyl-alanyl-D-glutamic acid: Diamino acid ligase of *Bifidobacterium globosum*. Arch. Microbiol..

[108] Zeng B., Wong K.K., Pompliano D.L., Reddy S., Tanner M.E. (1998). A phosphinate inhibitor of the meso-diaminopimelic acid-adding enzyme (MurE) of peptidoglycan biosynthesis. J. Org. Chem..

[109] Healy V.L., Lessard I.A., Roper D.I., Knox J.R., Walsh C.T. (2000). Vancomycin resistance in enterococci: Reprogramming of the d-Ala–d-Ala ligases in bacterial peptidoglycan biosynthesis. Chem. Biol..

[110] Miller D., Hammond S., Bugg T.H. (1998). Aminoalkylphosphinate inhibitors of D-Ala-D-Ala adding enzyme. J. Chem. Soc. Perkin Trans. 1.

[111] Mengin-Lecreulx D., van Heijenoort J., Park J.T. (1996). Identification of the mpl gene encoding UDP-N-acetylmuramate: L-alanyl-gamma-D-glutamyl-meso-diaminopimelate ligase in *Escherichia coli* and its role in recycling of cell wall peptidoglycan. J. Bacteriol..

[112] Hervé M., Boniface A., Gobec S., Blanot D., Mengin-Lecreulx D. (2007). Biochemical characterization and physiological properties of *Escherichia coli* UDP-N-acetylmuramate: L-alanyl-γ-d-glutamyl-meso-diaminopimelate ligase. J. Bacteriol..

[113] Doublet P., Van Heijenoort J., Bohin J.-P., Mengin-Lecreulx D. (1993). The murI gene of *Escherichia coli* is an essential gene that encodes a glutamate racemase activity. J. Bacteriol..

[114] Lundqvist T., Fisher S.L., Kern G., Folmer R.H., Xue Y., Newton D.T., Keating T.A., Alm R.A., de Jonge B.L. (2007). Exploitation of structural and regulatory diversity in glutamate racemases. Nature.

[115] Ashiuchi M., Yoshimura T., Esaki N., Ueno H., Soda K. (1993). Inactivation of glutamate racemase of Pediococcus pentosaceus with L-serine O-sulfate. Biosci. Biotechnol. Biochem..

[116] Tanner M.E., Miao S. (1994). The synthesis and stability of aziridino-glutamate, an irreversible inhibitor of glutamate racemase. Tetrahedron Lett..

[117] Sugio S., Kashima A., Kishimoto K., Peisach D., Petsko G.A., Ringe D., Yoshimura T., Esaki N. (1998). Crystal structures of L201A mutant of D-amino acid aminotransferase at 2.0 A resolution: Implication of the structural role of Leu201 in transamination. Protein Eng..

[118] Bugg T., Walsh C. (1992). Intracellular steps of bacterial cell wall peptidoglycan biosynthesis: Enzymology, antibiotics, and antibiotic resistance. Nat. Prod. Rep..

[119] Ha S., Walker D., Shi Y., Walker S. (2000). The 1.9 Å crystal structure of *Escherichia coli* MurG, a membrane-associated glycosyltransferase involved in peptidoglycan biosynthesis. Protein Sci..

[120] Bouhss A., Trunkfield A.E., Bugg T.D., Mengin-Lecreulx D. (2007). The biosynthesis of peptidoglycan lipid-linked intermediates. FEMS Microbiol. Rev..

[121] Breukink E., de Kruijff B. (2006). Lipid II as a target for antibiotics. Nat. Rev. Drug Discov..

[122] Kitagawa N., Otani T., Inai T. (2019). Nisin, a food preservative produced by Lactococcus lactis, affects the localization pattern of intermediate filament protein in HaCaT cells. Anat. Sci. Int..

[123] Kooy J. (1952). Strains of *Lactobacillus plantarum* which inhibit the activity of the antibiotics produced by *Streptococcus lactis*. Neth. Milk Dairy J..

[124] Maisnier-Patin S., Richard J. (1996). Cell wall changes in nisin-resistant variants of *Listeria innocua* grown in the presence of high nisin concentrations. FEMS Microbiol. Lett..

[125] Kramer N.E., van Hijum S.A., Knol J., Kok J., Kuipers O.P. (2006). Transcriptome analysis reveals mechanisms by which *Lactococcus lactis* acquires nisin resistance. Antimicrob. Agents Chemother..

[126] Hvorup R.N., Winnen B., Chang A.B., Jiang Y., Zhou X.F., Saier M.H. (2003). The multidrug/oligosaccharidyl-lipid/polysaccharide (MOP) exporter superfamily. Eur. J. Biochem..

[127] Inoue A., Murata Y., Takahashi H., Tsuji N., Fujisaki S., Kato J.-i. (2008). Involvement of an essential gene, mviN, in murein synthesis in *Escherichia coli*. J. Bacteriol..

[128] Ruiz N. (2008). Bioinformatics identification of MurJ (MviN) as the peptidoglycan lipid II flippase in *Escherichia coli*. Proc. Natl. Acad. Sci. USA.

[129] Kuk A.C., Mashalidis E.H., Lee S.-Y. (2017). Crystal structure of the MOP flippase MurJ in an inward-facing conformation. Nat. Struct. Mol. Biol..

[130] Zheng S., Sham L.-T., Rubino F.A., Brock K.P., Robins W.P., Mekalanos J.J., Marks D.S., Bernhardt T.G., Kruse A.C. (2018). Structure and mutagenic analysis of the lipid II flippase MurJ from *Escherichia coli*. Proc. Natl. Acad. Sci. USA.

[131] Kohga H., Mori T., Tanaka Y., Yoshikaie K., Taniguchi K., Fujimoto K., Fritz L., Schneider T., Tsukazaki T. (2022). Crystal structure of the lipid flippase MurJ in a “squeezed” form distinct from its inward-and outward-facing forms. Structure.

[132] Chu J., Vila-Farres X., Inoyama D., Ternei M., Cohen L.J., Gordon E.A., Reddy B.V.B., Charlop-Powers Z., Zebroski H.A., Gallardo-Macias R. (2016). Discovery of MRSA active antibiotics using primary sequence from the human microbiome. Nat. Chem. Biol..

[133] Kohga H., Lertpreedakorn N., Miyazaki R., Wu S., Hosoda K., Tanaka H., Takahashi Y.S., Yoshikaie K., Kuruma Y., Shigematsu H. (2025). Phage lysis protein LysM acts as a wedge to block MurJ conformational changes. Sci. Adv..

[134] Kuk A.C., Hao A., Lee S.-Y. (2022). Structure and mechanism of the lipid flippase MurJ. Annu. Rev. Biochem..

[135] Zhou P., Barb A.W. (2008). Mechanism and inhibition of LpxC: An essential zinc-dependent deacetylase of bacterial lipid A synthesis. Curr. Pharm. Biotechnol..

[136] Mochalkin I., Knafels J.D., Lightle S. (2008). Crystal structure of LpxC from Pseudomonas aeruginosa complexed with the potent BB-78485 inhibitor. Protein Sci..

[137] Tomaras A.P., McPherson C.J., Kuhn M., Carifa A., Mullins L., George D., Desbonnet C., Eidem T.M., Montgomery J.I., Brown M.F. (2014). LpxC inhibitors as new antibacterial agents and tools for studying regulation of lipid A biosynthesis in Gram-negative pathogens. mBio.

[138] Cordillot M., Dubée V., Triboulet S., Dubost L., Marie A., Hugonnet J.-E., Arthur M., Mainardi J.-L. (2013). In vitro cross-linking of *Mycobacterium tuberculosis* peptidoglycan by L, D-transpeptidases and inactivation of these enzymes by carbapenems. Antimicrob. Agents Chemother..

[139] de Munnik M., Lang P.A., Calvopiña K., Rabe P., Brem J., Schofield C.J. (2024). Biochemical and crystallographic studies of l, d-transpeptidase 2 from *Mycobacterium tuberculosis* with its natural monomer substrate. Commun. Biol..

[140] Pidgeon S.E., Apostolos A.J., Nelson J.M., Shaku M., Rimal B., Islam M.N., Crick D.C., Kim S.J., Pavelka M.S., Kana B.D. (2019). L, D-transpeptidase specific probe reveals spatial activity of peptidoglycan cross-linking. ACS Chem. Biol..

[141] Dik D.A., Marous D.R., Fisher J.F., Mobashery S. (2017). Lytic transglycosylases: Concinnity in concision of the bacterial cell wall. Crit. Rev. Biochem. Mol. Biol..

[142] Roney I.J., Rudner D.Z. (2023). Two broadly conserved families of polyprenyl-phosphate transporters. Nature.

[143] Oluwole A.O., Kalmankar N.V., Guida M., Bennett J.L., Poce G., Bolla J.R., Robinson C.V. (2024). Lipopeptide antibiotics disrupt interactions of undecaprenyl phosphate with UptA. Proc. Natl. Acad. Sci. USA.

[144] Kusuma K.D., Payne M., Ung A.T., Bottomley A.L., Harry E.J. (2019). FtsZ as an antibacterial target: Status and guidelines for progressing this avenue. ACS Infect. Dis..

[145] Nogales E., Downing K.H., Amos L.A., Löwe J. (1998). Tubulin and FtsZ form a distinct family of GTPases. Nat. Struct. Biol..

[146] Löwe J., Amos L.A. (1998). Crystal structure of the bacterial cell-division protein FtsZ. Nature.

[147] Kifayat S., Yele V., Ashames A., Sigalapalli D.K., Bhandare R.R., Shaik A.B., Nasipireddy V., Sanapalli B.K.R. (2023). Filamentous temperature sensitive mutant Z: A putative target to combat antibacterial resistance. RSC Adv..

[148] Konovalova A., Kahne D.E., Silhavy T.J. (2017). Outer membrane biogenesis. Annu. Rev. Microbiol..

[149] Schiffrin B., Brockwell D.J., Radford S.E. (2017). Outer membrane protein folding from an energy landscape perspective. BMC Biol..

[150] Gatsos X., Perry A.J., Anwari K., Dolezal P., Wolynec P.P., Likić V.A., Purcell A.W., Buchanan S.K., Lithgow T. (2008). Protein secretion and outer membrane assembly in *Alphaproteobacteria*. FEMS Microbiol. Rev..

[151] Miller R.D., Iinishi A., Modaresi S.M., Yoo B.-K., Curtis T.D., Lariviere P.J., Liang L., Son S., Nicolau S., Bargabos R. (2022). Computational identification of a systemic antibiotic for gram-negative bacteria. Nat. Microbiol..

[152] Srinivas N., Jetter P., Ueberbacher B.J., Werneburg M., Zerbe K., Steinmann J., Van der Meijden B., Bernardini F., Lederer A., Dias R.L. (2010). Peptidomimetic antibiotics target outer-membrane biogenesis in *Pseudomonas aeruginosa*. Science.

[153] Storek K.M., Auerbach M.R., Shi H., Garcia N.K., Sun D., Nickerson N.N., Vij R., Lin Z., Chiang N., Schneider K. (2018). Monoclonal antibody targeting the β-barrel assembly machine of *Escherichia coli* is bactericidal. Proc. Natl. Acad. Sci. USA.

[154] Rohmer M., Knani M., Simonin P., Sutter B., Sahm H. (1993). Isoprenoid biosynthesis in bacteria: A novel pathway for the early steps leading to isopentenyl diphosphate. Biochem. J..

[155] Rohmer M., Grosdemange-Billiard C., Seemann M., Tritsch D. (2004). Isoprenoid biosynthesis as a novel target for antibacterial and antiparasitic drugs. Curr. Opin. Investig. Drugs.

[156] Jansson A.M., Wieckowska A., Björkelid C., Yahiaoui S., Sooriyaarachchi S., Lindh M., Bergfors T., Dharavath S., Desroses M., Suresh S. (2013). DXR inhibition by potent mono-and disubstituted fosmidomycin analogues. J. Med. Chem..

[157] Bem A.E., Velikova N., Pellicer M.T., Baarlen P.v., Marina A., Wells J.M. (2015). Bacterial histidine kinases as novel antibacterial drug targets. ACS Chem. Biol..

[158] Rosales-Hurtado M., Meffre P., Szurmant H., Benfodda Z. (2020). Synthesis of histidine kinase inhibitors and their biological properties. Med. Res. Rev..

[159] Sauvage E., Kerff F., Terrak M., Ayala J.A., Charlier P. (2008). The penicillin-binding proteins: Structure and role in peptidoglycan biosynthesis. FEMS Microbiol. Rev..

[160] Nicola G., Tomberg J., Pratt R., Nicholas R.A., Davies C. (2010). Crystal structures of covalent complexes of β-lactam antibiotics with *Escherichia coli* penicillin-binding protein 5: Toward an understanding of antibiotic specificity. Biochemistry.

[161] Nicola G., Peddi S., Stefanova M., Nicholas R.A., Gutheil W.G., Davies C. (2005). Crystal structure of *Escherichia coli* penicillin-binding protein 5 bound to a tripeptide boronic acid inhibitor: A role for Ser-110 in deacylation. Biochemistry.

[162] Eschenburg S., Priestman M., Schönbrunn E. (2005). Evidence that the fosfomycin target Cys115 in UDP-N-acetylglucosamine enolpyruvyl transferase (MurA) is essential for product release. J. Biol. Chem..

[163] Ogris I., Zupančič B., Sosič I., Merzel F., Grdadolnik S.G. (2025). Mechanistic insight into the dynamics of Mur ligase through a comprehensive timescale-specific approach. Commun. Chem..

[164] Sullivan G.J., Delgado N.N., Maharjan R., Cain A.K. (2020). How antibiotics work together: Molecular mechanisms behind combination therapy. Curr. Opin. Microbiol..

[165] Avery L.M., Nicolau D.P. (2018). Investigational drugs for the treatment of infections caused by multidrug-resistant Gram-negative bacteria. Expert Opin. Investig. Drugs.

[166] Appelbaum P.C., Jacobs M.R. (2005). Recently approved and investigational antibiotics for treatment of severe infections caused by Gram-positive bacteria. Curr. Opin. Microbiol..

[167] Terreni M., Taccani M., Pregnolato M. (2021). New antibiotics for multidrug-resistant bacterial strains: Latest research developments and future perspectives. Molecules.

